# Multi-reporter selection for the design of active and more specific zinc-finger nucleases for genome editing

**DOI:** 10.1038/ncomms10194

**Published:** 2016-01-07

**Authors:** Benjamin L. Oakes, Danny F. Xia, Elizabeth F. Rowland, Denise J. Xu, Irina Ankoudinova, Jennifer S. Borchardt, Lei Zhang, Patrick Li, Jeffrey C. Miller, Edward J. Rebar, Marcus B. Noyes

**Affiliations:** 1 The Lewis-Sigler Institute for Integrative Genomics, Princeton University, Princeton, New Jersey 08544, USA; 2 Sangamo BioSciences Inc., Richmond, California 94804, USA; 3 Department of Molecular Biology, Princeton University, Princeton, New Jersey 08544, USA; 4 Institute for Systems Genetics, NYU School of Medicine, New York, New York 10016, USA; 5 Department of Biochemistry and Molecular Pharmacology, NYU School of Medicine, New York, New York 10016, USA

## Abstract

Engineered nucleases have transformed biological research and offer great therapeutic potential by enabling the straightforward modification of desired genomic sequences. While many nuclease platforms have proven functional, all can produce unanticipated off-target lesions and have difficulty discriminating between homologous sequences, limiting their therapeutic application. Here we describe a multi-reporter selection system that allows the screening of large protein libraries to uncover variants able to discriminate between sequences with substantial homology. We have used this system to identify zinc-finger nucleases that exhibit high cleavage activity (up to 60% indels) at their targets within the *CCR5* and *HBB* genes and strong discrimination against homologous sequences within *CCR2* and *HBD*. An unbiased screen for off-target lesions using a novel set of *CCR5*-targeting nucleases confirms negligible *CCR2* activity and demonstrates minimal off-target activity genome wide. This system offers a straightforward approach to generate nucleases that discriminate between similar targets and provide exceptional genome-wide specificity.

## Abstract (web summary)

Zinc finger nucleases have an established role in genome editing. Here, the authors report a strategy for identifying zinc finger nucleases that discriminate between desired targets and provide genome-wide specificity.

## Graphical abstract

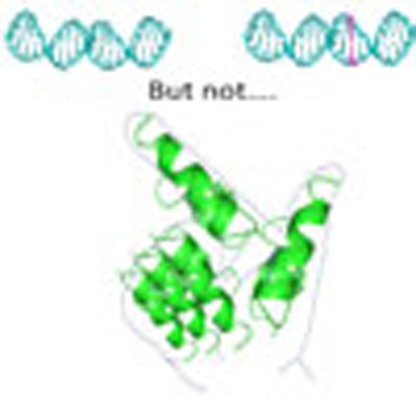

Designer nucleases have revolutionized studies of gene structure and function in higher organisms by enabling the simple and efficient manipulation of specified genomic sequences^1^. While several different nuclease platforms have been developed for this purpose, the strategy they employ is the same: direct a double-strand break (DSB) to a desired genomic locus and allow repair mechanisms to edit the sequence. If the break is repaired by mutagenic non-homologous end joining (NHEJ), the insertions or deletions (indels) that often result can lead to frame shifts and functional knockouts^2^. If the break is repaired by homology-directed repair (HDR), the sequence can be rewritten if an appropriate donor template of DNA is provided^3^,^4^. This approach has proven successful across diverse species and platforms^5^,^6^,^7^,^8^,^9^,^10^,^11^,^12^,^13^.

As applications for designer nucleases have multiplied and extended into more sensitive areas such as stem cell biology and therapeutics, the focus of design efforts has shifted from improving activity to optimizing genome-wide specificity^14^,^15^,^16^,^17^,^18^,^19^. A key element of this shift has been the emerging realization that nucleases designed for genome-editing applications will frequently need to discriminate against highly similar off-target sequences, due to the high frequency of repeat elements, gene duplications and pseudogenes in eukaryotic genomes. Further complicating matters, successful HDR can be limited by the distance between the DSB and the sequence to be modified^20^. For these reasons, the precise location of a DSB may be critical, and similarity with potential off-target sequences unavoidable.

The crux of a high-fidelity designer nuclease are the abilities to both strongly specify a desired sequence and to avoid others. While strong *in vivo* on-target activity has been demonstrated for all the designer nuclease platforms (zinc-finger nucleases (ZFNs), homing endonucleases, transcription activator-like effector domains (TALENs) and RNA-guided CRISPR-Cas9 systems (RGENs)), fidelity remains uncertain. Early-stage ZFNs^21^,^22^, TALENs^23^,^24^,^25^ and RGENs^24^,^25^,^26^,^27^,^28^ can, in certain cases, show substantial activity at alternative sequences similar to the designed target as well as unanticipated sequences. Several approaches have been employed to address these issues. Nicking RGENs^18^, Cas9-Fok1 fusions^29^,^30^, RNA guide length^19^ and alternative TALE domains^17^ have all been employed to address concerns of off-target activity. Furthermore, algorithms that allow the user to choose a specific target sequence based on its reduced risk of off-target activity have been developed^31^,^32^,^33^. However, these approaches limit therapeutic applications that require precise modification of sequences with high degrees of homology to other regions of the genome.

To discriminate between similar targets, a complex protein–DNA interface with great engineering potential, coupled with a powerful selection system for identifying new designs that exhibit desired binding properties, may be required. Therefore, the complexities of intra- and inter-finger interactions offered by the zinc-finger domain^34^,^35^,^36^,^37^ may provide advantages over the more modular TALEN and RGEN platforms. However, until recently diverse pools of zinc fingers able to bind all 3-nt (nucleotide) targets were unavailable, and few selection systems allowed for the recovery of proteins with discriminatory attributes. Prior ZFN engineering for *in vivo* application by both bacterial one^38^ and two-hybrid^39^,^40^ assays have been primarily limited to pools of 5′-GNN-3′ binding zinc fingers and do not present a counter-selective element that would produce zinc fingers able to discriminate between closely related sequences. Therefore, this new complete zinc-finger pool set coupled with a system that provides designer nucleases, zinc finger or otherwise, that can distinguish between two closely related sequences would be of great utility.

We recently described a new resource of zinc-finger pools with significantly greater depth than previously available^41^. Here we describe a multi-reporter selection system to screen libraries of these zinc fingers for combinations that can discriminate between two similar targets. We apply this system to identify ZFNs that provide excellent discrimination between highly homologous sequences for two targets *in vivo*: CCR5 versus CCR2 and haemoglobin beta (HBB) versus haemoglobin delta (HBD). In each case we recover ZFN pairs that manifest strong on-target activity with minimal or no detectible activity at the homologous off-target sequence. Finally, in an unbiased screen of genome-wide off-target activity for a subset of the CCR5 ZFN pairs, we identify just a small number of low-frequency off-target loci. For one of these ZFN pairs, we identify just a single off-target locus modified above 0.1% (0.37% lesion frequency at this locus) demonstrating exceptional genome-wide fidelity. This work demonstrates the ability of the multi-reporter selection system to uncover proteins with fine-tuned specificity and the potential for pristine genome editing.

## Results

### Establishment of a multi-reporter selection system

The omega-based bacterial one-hybrid (B1H) system has proven a simple and extremely sensitive method for the investigation of protein–DNA interactions^38^,^42^,^43^,^44^,^45^,^46^ (see overview, Supplementary Note 1). This system differentiates itself from other bacterial hybrid assays through the employment of the omega subunit of RNA polymerase (*rpoZ*) as the fusion partner to the protein of interest. In this way omega acts as an activation domain through recruitment of the polymerase. Omega is a non-essential component of the core holoenzyme^47^,^48^ allowing selections to be carried out in an *rpoZ* knockout strain and therefore in the absence of competition from endogenous omega. The lack of competition allows activation of the reporter even at low levels of fusion protein expression and the recovery of interactions with a large range of affinities^42^. As a result, the method has allowed the characterization of transcription factor DNA-binding specificities for most common DNA-binding domain families^42^,^45^,^49^,^50^ as well as the selection of synthetic homeodomains^43^ and zinc fingers^38^,^41^,^44^ with novel specificities.

**Figure 1:**
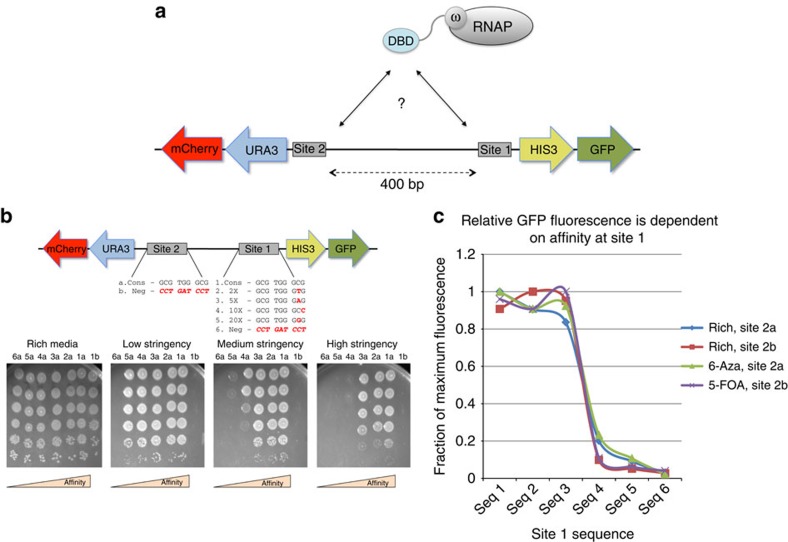
Multiple reporters allow the simultaneous investigation of independent interactions by bacterial hybrid assay. (**a**) Rearrangement of the original B1H reporter vector to express the selectable HIS3 and URA3 markers from separate promoters allows the assay of two independent interactions simultaneously. The addition of fluorescent markers mCherry and GFP provides a secondary, graded measure of activity for each interaction. (**b**) Survival is dependent on the affinity of the protein–DNA interaction and the selection conditions that impact each reporter independently. Here *Zif268* is expressed as an omega fusion. *Zif268*'s consensus binding site (labelled a) is placed in front of the URA3 reporter and paired with one of a set of binding sites of known affinity in front of HIS3 (numbered from 1 to 6 by the noted ‘X'-fold decrease in affinity offered by each site^51^). As a control, a sequence *Zif268* will not bind to (labelled b) is placed in front of URA3 and paired with the consensus in front of HIS3. Log-phase cells were titred in 10-fold dilutions from top to bottom on rich media or selective plates that contain 6-azauracil and either a low (2 mM), medium (5 mM) or high (20 mM) level of 3-AT. Survival is dependent on activation of URA3 (1a versus 1b) and related to the affinity of the interaction that drives HIS3 expression. (**c**) Fluorescent output is related to the affinity of the protein–DNA interaction that drives its expression and unrelated to the selection conditions impacting the competing binding site and reporter. The same cells tested in **c**, as well as a complete set of the site 1 sequences paired with the negative ‘b' sequence, were grown in either rich media or selective media that impacts only URA3 expression. While HIS3 expression is not selected for, GFP output is related to the affinity of the interaction that drives the HIS3/GFP reporter and unrelated to the growth conditions that impact site 2 and URA3/mCherry.

For these studies, we improved this method and adapted it to the challenge of providing highly effective DNA-binding proteins that could discriminate homologous sequences. To accomplish this, we first created a variation of the B1H system in which its two selectable markers (HIS3 and URA3; Fig. 1a) are expressed from independent promoters. Our goal in doing this was to enable simultaneous selection for or against activation of these two reporter genes. To test whether our new system accomplished this, we compared the activity of the reporters while varying known protein–DNA interactions that drive their expression. Here we expressed the three-finger *Zif268* zinc-finger protein as a direct fusion to omega. We fixed the *Zif268* consensus target sequence upstream of the promoter that drives the URA3 reporter and paired this with alternative *Zif268* targets^51^ that exhibit a range of affinities upstream of the HIS3 reporter. Bacteria bearing each *Zif268*-binding site combination were grown to log phase, titred and plated on selective media. Only cells that offered a functional interaction to drive URA3 survived the presence of 6-azauracil (6-aza) while the affinity of the interaction that drives HIS3 determined survival at various stringency 3-amino triazole (3-AT) concentrations (Fig. 1b). The reverse of this is also true when the sequences in sites 1 and 2 are switched (Supplementary Fig. 1). We confirmed these observations in follow-up studies of doubling time in liquid media (Supplementary Fig. 2). In sum, while maintaining a desired protein–DNA interaction that drives one reporter, the interaction that drives the secondary reporter can be selected for, or against, through the addition of inhibitors in the media.

By separating the HIS3 and URA3 reporters we are able to investigate two independent interactions simultaneously. Still, survival alone does not allow us to differentiate between the attributes of multiple, functional interactions, only indicating that they all surpass the survival threshold required of the selection. Therefore, as a second improvement to the original B1H system, we added fluorescent reporters to each of the selectable markers to provide a secondary and more graded measure of activity (henceforth referred to as the multi-reporter bacterial one-hybrid system (MR-B1H)). A green fluorescent protein (GFP) cassette is expressed from the same promoter that drives HIS3 expression, and mCherry expressed from the promoter that drives URA3 (Fig. 1a). To demonstrate the utility of this approach, we again turned to *Zif268*. While fixing the interaction that drives URA3 expression, we tested the same set of binding sites as above to drive HIS3 and therefore, GFP expression (Fig. 1c). With this system we observed that under varying conditions of URA3 selection (for, against or neutral), mCherry expression is related to the selection conditions but is otherwise constant (Supplementary Fig. 3). We also found that, as expected, GFP expression is related to the affinity of the protein–DNA interaction that drives the HIS3/GFP promoter and unrelated to the URA3-focused experimental conditions (Fig. 1c). These results confirm that selection conditions designed to influence URA3 expression do not impact the HIS3/GFP transcription. Therefore, activation of the reporter is a function of the protein–DNA interaction that drives its expression and the fluorescent output is a secondary measure of the activity that interaction offers.

### Selection of proteins by target discrimination

Having shown that that the MR-B1H system can function as a reporter to compare the relative strengths of two interactions, we next sought to demonstrate that this system can be used to select proteins with new binding properties. We first prepared libraries of four-finger zinc-finger DNA-binding proteins using our recently reported, complete set of selected zinc-finger pools^41^ as templates for library assembly (Supplementary Fig. 4). This approach was guided by prior strategies for assembly of zinc-finger pools^38^,^52^,^53^,^54^. By selecting four-finger variants from diverse pool assemblies we circumvent inter-finger complications that often arise when neighbouring fingers of known specificity are designed next to one another. Rather, we select combinations of zinc fingers from four-finger libraries that are most compatible with one another.

**Figure 2:**
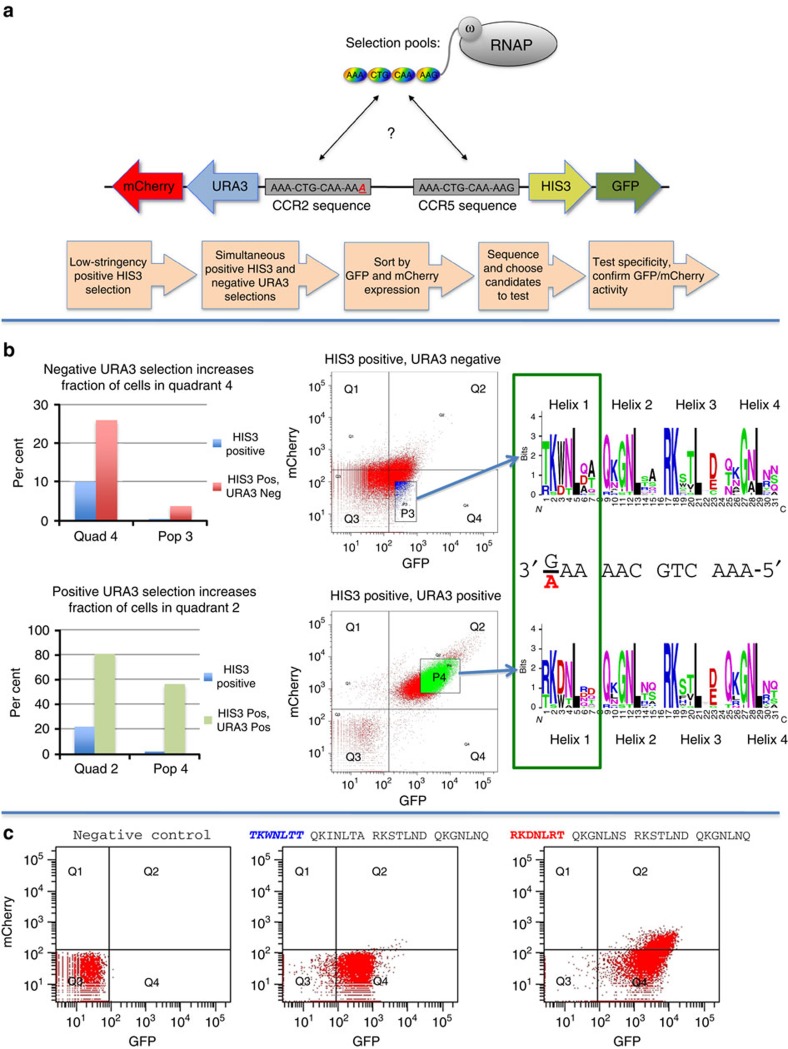
Selection of zinc fingers that can discriminate between similar targets. (**a**) Diagram depicts the selection process. The desired and counter targets are placed in front of the promoters that drive HIS3 and URA3 expression, respectively. Zinc-finger pools previously selected to bind each 3 bp subsite of the desired target are used as PCR templates to assemble a four-finger library, illustrated as rainbow-coloured ovals. This four-finger library is expressed as an omega fusion. To select four-finger members of this library, which are able to discriminate between the desired targets, cells are grown under conditions that are inhibited by URA3 expression but required HIS3 activation. A workflow of the procedure is shown below. (**b**) Selection conditions influence the enriched amino acids that correspond to the target mismatch. Using the library described in **a**, selection for HIS3 activation but against URA3 activation increases the fraction of the population in the GFP-positive (Pos), mCherry-negative (Neg) quadrant 4 in comparison with a HIS3-positive selection alone (top). Using the same library, selection for both HIS3 and URA3 activations increases the fraction of the population in the GFP-positive, mCherry-positive quadrant 2 in comparison to a HIS3-positive selection alone (bottom). Sequencing the zinc fingers recovered from stringent populations of these selection conditions reveal a stark difference in the amino acids enriched in the helix that corresponds to the difference in the desired and counter target (green box). (**c**) The binding attributes are confirmed for zinc-finger candidates that represent the recovered pools in **b**. Zinc-finger candidates are paired with the CCR5 versus CCR2 reporter vector and grown without selection. The GFP versus mCherry attributes are complementary to the selection conditions from which the candidate protein was derived. The sequence of the tested zinc-finger helices are shown (N to C) above the dot plots with the discriminator helix shown in colour.

To expand the utility of the MR-B1H system, we screened our libraries, created from our individual pools, to uncover proteins able to discriminate between related sequences. For our initial study, we targeted a well-characterized sequence within the *CCR5* gene, at which indels can mediate efficient functional inactivation and cellular resistance to HIV infection^22^,^55^,^56^,^57^. This locus is targeted by a ZFN dimer currently in clinical studies. This target provided an attractive initial test of our selection system, since it exhibits substantial sequence identity with a second sequence in the human genome (within the homologous *CCR2* gene), and the availability of highly active and specific published reagents^22^ for this target would provide a benchmark against which to gauge the performance of any selected ZFNs. To utilize this tool, we modified the MR-B1H system to select zinc-finger arrays able to bind the CCR5 target and provide discrimination against the CCR2 target (Fig. 2a).

The 12-nt sequence targeted by the published 3′ CCR5 ZFN monomer^22^ (right target) was installed upstream of the HIS3/GFP reporter (Fig. 2a). The homologous CCR2 sequence (matching at 11/12 bases) was installed upstream of the URA3/mCherry reporter. ZFP array libraries were expressed as omega fusions and paired with this CCR5-focused MR-B1H reporter vector (Supplementary Fig. 4). A low-stringency, HIS3-positive selection was performed to remove non-functional arrays from the library. The surviving library members were pooled, again paired with the reporters and selected for activation of HIS3 (CCR5 target), with a secondary selection for, against or neutral for URA3 (CCR2 target). Classification of these cells as GFP or mCherry positive was defined by setting gates to established background-level fluorescence using a negative control that should not actively express either fluorescent reporter (see Fig. 2c as an example). With experimental samples, when activation of URA3 is selected against, the number of cells above the GFP background but below the mCherry threshold increased by 2.5-fold over cells grown with HIS3 selection alone (Fig. 2b, top, quadrant 4). Moreover, these conditions produced a stringent population of high GFP and low mCherry activities (Fig. 2b, pop 3) enriched by 10-fold. Conversely, when activation of URA3 is selected for, 80% of the enriched cells are both GFP and mCherry positive (Fig. 2b, bottom), a 4-fold enrichment over the HIS3 selection alone. Populations of these cells were recovered and the ZFPs they harbour were sequenced (Fig. 2b, right). The amino acids enriched in the alpha helix of the N-terminal finger are noticeably different depending on the URA3 selection conditions (Fig. 2b, green box). On the basis of the canonical model of ZFP–DNA recognition, this first helix should bind to the AAG and AAA triplets that differentiate BS1 from BS2 of the reporter, respectively. Candidate ZFPs that represent these populations were tested again with the reporters to confirm the fluorescent activity, and thus their DNA-binding attributes, in the absence of selective pressure (Fig. 2c). These studies confirmed that we can select new ZFP arrays from combinatorial libraries that offer fine-tuned attributes by modifying selection conditions and recovering fluorescent populations that represent the characteristics we desire.

### Fine-tuned ZFNs improve CCR5–CCR2 discrimination *in vivo*

**Figure 3:**
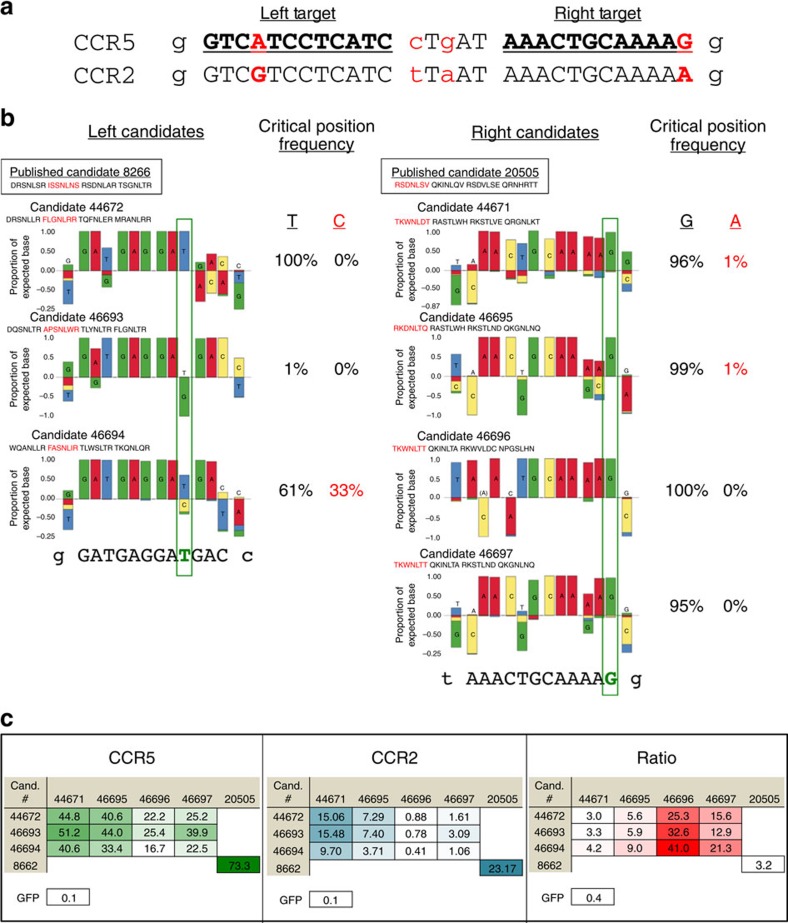
MR-B1H-produced zinc fingers provide high CCR5 activity with strong discrimination against CCR2. (**a**) The corresponding CCR5 versus CCR2 sequences are shown with the 12-bp zinc-finger targets bold and underlined. Sequences are shown 5′ to 3′, but because ZFN monomers bind opposite strands of DNA, the left ZFN monomer targets the reverse complement of the sequence shown (compare with target in **b**). Mismatches with the CCR2 sequence are shown as red letters. (**b**) SELEX results for candidate zinc fingers. Candidates' (Cand.) identification numbers and the sequences of their helices are listed above each SELEX plot. Helices that correspond to the discriminatory base are shown in red. The discriminatory base is boxed in green. The recovered percentage of sequences that correspond to the CCR5 or CCR2 base at this critical position are listed to the right of each plot. Those that tolerate binding to the selected-against base are shown as red numbers. The previously published helices for each left and right monomers are boxed at the top. (**c**) Candidate ZFN pairs were expressed *in vivo* and the percentage of indels at CCR5 and CCR2 measured (left and middle tables). The published candidates 8266–20505 were tested in parallel for reference. The ratio of CCR5 to CCR2 indel frequency is shown in the right table. The indel frequency recovered from a control that expressed GFP rather than a nuclease pair is shown below.

The MR-B1H system is able to provide ZFPs that function with fine-tuned specificity in *Escherichia coli*, however, our goal is to provide target discrimination in human cells. To test our selected ZFPs outside of bacteria, we first repeated the procedure above to select ZFPs able to bind the published 5′ CCR5 target (left target) while discriminating against the CCR2 sequence (Supplementary Fig. 5). Next, we assessed the DNA-binding specificity of a set of selected ‘left' and ‘right' ZFPs using systematic evolution of ligands by exponential enrichment (SELEX). For this study, candidates were chosen as intact proteins rather than designed from a consensus to preserve context-dependent aspects of performance. As shown in our previous studies^41^ all amino acids within a recognition helix may influence one another. Therefore, we took a conservative approach and chose only complete, selected ZFPs to follow-up in all experiments reported here. The SELEX results revealed generally good specificity by the selected ZFPs, with several exhibiting a marked preference for the intended target base at those positions differing between CCR5 and CCR2 (Fig. 3a,b). Next, we expressed these candidates as ZFN pairs in K562 cells and quantified their activity at the CCR5 and CCR2 targets (Fig. 3c). For comparison, a published set of CCR5-targeted ZFNs^55^ were tested in parallel with the MR-B1H-produced ZFNs. We found that our selected ZFNs were highly active, yielding up to 50% indels at CCR5. Moreover, many were also highly selective for CCR5 versus CCR2, with half yielding a modification ratio of >12. Interestingly, while the left monomers have some influence, the CCR2 activity of the MR-B1H-produced ZFNs appears to be primarily related to the specificity of the right monomer and its ability to bind the adenine that differentiates CCR2 from CCR5 (see SELEX results, Fig. 3b). Those with no SELEX evidence of binding adenine at this position (candidates 46696 and 46697) offer CCR5 to CCR2 indel ratios that range from 12.9 to 41. For comparison, under these experimental conditions the previously published ZFNs yielded 73% modification of CCR5 in this study, with a threefold preference versus cleavage of CCR2.

### Extending fine-tuned ZFNs reduces competitive activity

The MR-B1H system can uncover ZFPs with strong discrimination between two targets that differ by a single base pair, even when we have restricted ourselves to duplicate an exact target in the literature. However, with a complete C2H2 zinc-finger pool set we are not limited by sequence and have the flexibility to slightly shift and expand the zinc-finger target, if advantageous, and still remain in close proximity to the sequence to be modified. Therefore, we reasoned that by increasing the number of fingers per monomer and maximizing the counter selection by focusing on the divergence between homologous targets, we could further improve the discrimination that our ZFNs are able to offer.

**Figure 4:**
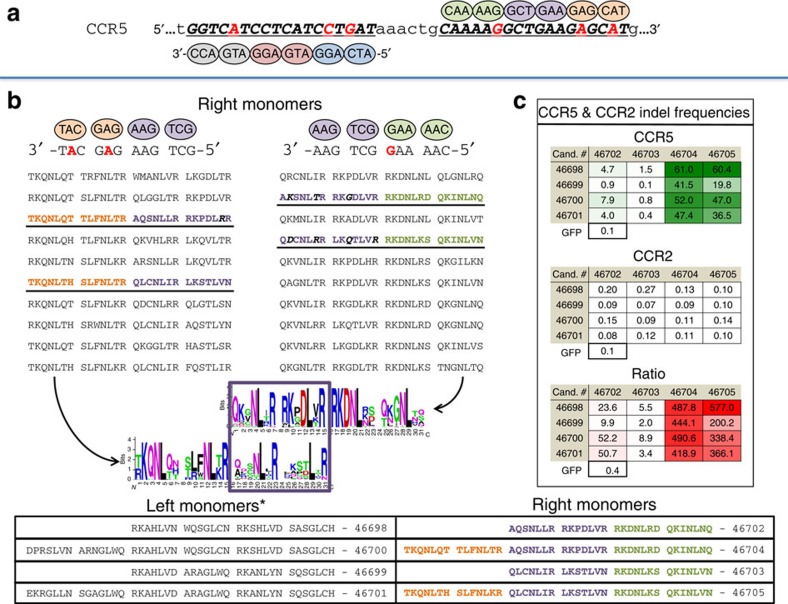
Extending zinc-finger targets to increase CCR2 discrimination. (**a**) The CCR5 target is shifted 6 nt 3′ relative to the target in Fig. 3 (bold and underlined). Mismatches with the CCR2 sequence are shown as red letters. Each ZFN monomer is increased from four to six fingers. (**b**) For each target, two overlapping four-finger libraries are produced. The right monomer pools are shown here while the left monomer pools are shown in Supplementary Fig. 7. Pools of these libraries are colour coded to emphasize that the overlapping zinc fingers target the same sequences. Zinc fingers are selected from each pool by selection for the CCR5 sequence but against the CCR2 sequence. Targets are shown 3′ to 5′ to emphasize the overlap in the targets of the four-finger selections. From each of these selections, 10 of the selected ZFPs are shown. Candidates (Cand.) used to design the four- and six-finger monomers employed as nucleases are bold and underlined. All enriched amino acids for each of the four-finger selections are shown below as a sequence logo with the overlapping two fingers boxed in purple. (**c**) Candidate ZFN pairs were expressed *in vivo* and the percentage of indels at CCR5 and CCR2 measured. Indel frequencies recovered at either target from cells that did not express a nuclease (GFP) are shown below each table. The ratio of CCR5 to CCR2 indel frequency is shown below. A table of the four- and six-finger zinc-finger helices used in the nuclease studies, shown N term to C term, is provided.

To test this approach we shifted the CCR5 target 6 bp 3′ (Supplementary Fig. 6). We also increased the number of mismatches between the CCR5 and CCR2 targets by extending each ZFP to contain six fingers per monomer (Fig. 4a and Supplementary Fig. 7). To limit library size and maintain the complexity for each finger, zinc fingers were selected from two overlapping four-finger libraries to bind these targets (selected ZFPs shown in Fig. 4b and Supplementary Fig. 7B). In this way, the two C-terminal and two N-terminal fingers of the overlapping libraries recognize the same sequences (purple ovals in Fig. 4b). Common fingers recovered in the ‘overlap positions' of both libraries were used as guides to design six-finger proteins. However, four-finger proteins recovered to bind the 12-nt target proximal to the cut site can also be used directly. In this way, both four- and six-finger proteins were selected using the counter-selection assay detailed above.

Two four- and two six-finger proteins that target the shifted CCR5 sequence were tested for their function as ZFNs in K562 cells. These ZFNs are related in that the four C-terminal fingers of the six-finger proteins are identical to one of the four-finger variants (see Fig. 4, table). Therefore, any differences in activity are due to the addition of two N-terminal fingers. As above, the indel frequencies at both the CCR5 and CCR2 loci were measured for each ZFN pair (Fig. 4c). Interestingly, the number of fingers has a large impact on the right target but not the left. Only ZFNs that utilize right monomers with six fingers provide high CCR5 indel frequencies, ranging from 19 to 61%. What is more, for each of these eight combinations the CCR2 indel frequency ranges from 0.09 to 0.14%, similar to the background frequency found from a GFP-transfected control sample. As a result, we are able to leverage differences in the CCR5 and CCR2 sequences, while remaining in close proximity to the desired cut site, by slightly shifting and expanding our target. By doing so we have created ZFNs that offer strong CCR5 activity and little if any activity at CCR2.

**Figure 5:**
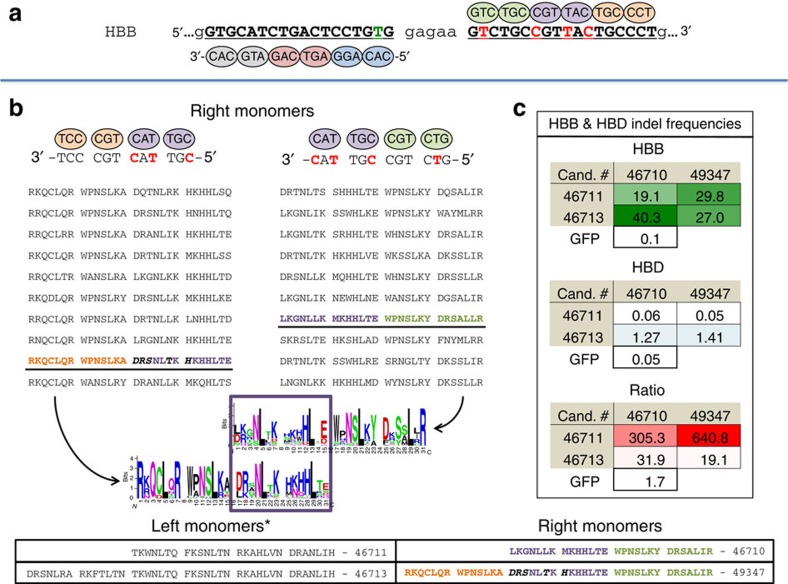
MR-B1H-produced zinc fingers provide high HBB activity with strong discrimination against HBD. (**a**) The HBB target is shown, mismatches to the HBD sequence are shown as red letters. The sickle cell causing mutation that separates the left target here from the HBD sequence is shown as a green letter. (**b**) As in Fig. 4, for each target, two overlapping four-finger libraries are produced. The right monomer pools are shown here while the left monomer pools are shown in Supplementary Fig. 8. Pools of these libraries are colour coded to emphasize that the overlapping zinc fingers target the same sequences. Zinc fingers are selected from each pool by selection for the HBB sequence but against the HBD sequence. Targets are shown 3′ to 5′ to emphasize the overlap in the targets of the four-finger selections. From each of these selections, 10 of the selected ZFPs are shown. Candidates (Cand.) used to design the four- and six-finger monomers employed as nucleases are bold and underlined. All enriched amino acids for each of the four-finger selections are shown below as a sequence logo with the overlapping two fingers boxed in purple. (**c**) Candidate ZFN pairs were expressed *in vivo* and the percentage of indels at HBB and HBD measured. Indel frequencies recovered at either target from cells that did not express a nuclease (GFP) are shown below each table. The ratio of HBB to HBD indel frequency is shown below. A table of the four- and six-finger zinc-finger helices used in the nuclease studies, shown N term to C term, is provided.

A second disease-related target complicated by the need for discrimination against a highly conserved homologue is HBB. Mutations in the HBB sequence lead to sickle cell anaemia as well as other blood-borne diseases. The coding sequences for HBB and HBD are 93% identical in human making it difficult to modify HBB without altering HBD. Using the same approach as outlined above, we focused the selection of zinc fingers to bind directly at a mutant HBB sequence that causes sickle cell anaemia (Fig. 5a and Supplementary Fig. 8).

Overlapping four-finger libraries were created to select four-finger zinc fingers, and thereby design six-finger proteins that can discriminate between HBB and HBD using the same approach detailed above (selected ZFPs shown in Fig. 5b and Supplementary Fig. 8B). From these results, a four- and six-finger protein that bind the right and left targets were paired (see Fig. 5, table) and tested as ZFNs in K562 cells. For each pair, indel frequencies were measured at both the HBB and HBD loci (Fig. 5c). Interestingly, while these ZFNs produce high HBB indel frequencies regardless of finger number, the HBD indel frequency is largely dependent on the number of fingers in the left monomer. In both cases, the six-finger left monomers increase HBD indel frequencies from background levels to low percentages. As the extension of the left monomer does not pick up additional differences between the HBB and HBD sequences, it is possible the extended recognition increases the affinity at the HBD target while lessoning the consequence of the single mismatch present in the left monomer targets. These results imply that the extension of a nuclease target that does not increase the number of mismatches relative to a similar sequence in the genome may lead to an increase in off-target activity. Regardless, we have produced ZFNs that target the sickle cell mutation in HBB with high activity and in some cases, background levels of HBD activity.

### Unbiased genomic screen shows negligible off-target activity

The ability of a ZFP to discriminate between closely related sequences is a powerful indicator of *in vivo* specificity but does not eliminate the possibility that these fine-tuned nucleases bind and modify other, less-predictable sequences. This is apparent from unbiased screens of genome-wide cutting for ZFNs^22^, TALENs^24^,^25^ and RGENs^25^,^28^, which in some cases have revealed cleavage at sequences that are more diverged from the desired target relative to other, uncleaved sequences, demonstrating that genome-wide fidelity can be more complicated than sequence similarity alone. Therefore, to map off-target loci for our different CCR5 ZFN pairs, we used a modified version of this integrase-defective lentiviral vector (IDLV) capture and mapping protocol, which we have previously described^22^. To increase the sensitivity of the assay, we used the high sequencing depth provided by Illumina sequencing and performed the assay in biologic triplicate to minimize apparent clusters of IDLV integrations caused simply by obtaining more sequence reads. We ranked clusters of IDLV integrations (defined as IDLV integrations within 1,000 bp of other IDLV integrations) based on the total number of integrations, the number of replicates containing those integrations and the ratio of integrations in the ZFN-treated samples to the control samples (see Methods). We then designed PCR primers to amplify the top 20 ranked clusters for each ZFN pair and characterized the indel frequency in K562 cells at each of these 20 potential off-target sites per ZFN pair; to provide additional information we also characterized indel frequencies at loci corresponding to the top 20 ranked clusters for other CCR5 ZFNs that target similar sequences (that is, we tested the activity of 46698:46705 at clusters obtained with either 46698:46705 or 46700:46705). For the previously described 8266:20505 ZFN pair, this process revealed nine active off-target sites with an aggregate activity of 33.3% (Table 1). For the two new ZFN pairs that targeted the same sequence, 46693:46696 and 46693:46697, this process yielded 11 active off-target sites with an aggregate activity of 21.2% and 10 active off-target sites with an aggregate off-target activity of 6.6%, respectively. However, it is difficult to discern whether the decrease in aggregate off-target activity is due to improved genome-wide specificity compared with the previously described ZFN pair or simply a reflection of their lower overall activity under these conditions (Table 1).

The specificity of the new CCR5 ZFN pairs that target a slightly shifted and extended sequence, 46698:46705 and 46700:46705, allow a more meaningful comparison because they have similar activity to the previously described ZFN pair at the intended CCR5 site. For 46698:46705, four active off-target sites were identified with an aggregate activity of 1.5% (Table 2). The best results were obtained with 46700:46705 that had three active off-target sites and an aggregate activity of 0.5% (Table 2). This represents 22- and 67-fold decreases in aggregate off-target activity, respectively, relative to the published CCR5 ZFNs. Together these results indicate that the MR-B1H selection system allows the recovery of zinc fingers that can distinguish between homologous targets and at least in this case, offer excellent genome-wide fidelity. This stringent level of specificity was further supported by SELEX (Supplementary Fig. 9).

## Discussion

Here we describe the development of a multi-reporter selection system that enables the assay and selection of zinc fingers able to discriminate between similar targets. Zinc fingers were selected and employed as nucleases in human cells to differentiate between the *CCR5* and *CCR2* genes, as well as the *HBB* and *HBD* should be italicized in this context genes. These ZFNs provide significant discrimination between these targets offering strong on-target activity and in many cases negligible activity at the designed against off-target sequence. For the most promising CCR5 candidates an unbiased screen of genome-wide nuclease activity uncovered only a small number of low-frequency off-targets and in the best case, aggregate off-target activity of just 0.5%.

A number of other strategies for improving the cellular specificity of targeted nucleases have also been developed, including the use of dual nickases^18^, alternative delivery procedures^58^,^59^ and target-choosing algorithms^26^,^60^,^61^ that minimize homology between the target site and the rest of the genome. While strategies to measure off-target activity genome wide have been developed^22^,^58^,^59^ and demonstrate unanticipated levels of off-target activity in some nuclease systems, previously described specificity optimization approaches have generally been tested at limited numbers of bioinformatically predicted off-target sites and still failed to completely abolish off-target activity. Given this, it seems likely that ultimate specificity will require the application of multiple orthogonal approaches. We note that our strategy for improving specificity—selection-based engineering of the protein–DNA interface—may be combined with these other methods. In this work, however, to demonstrate the utility of our methods we have deliberately avoided combining approaches and have taken on a ‘worst case scenario' selecting nucleases to target sequences complicated by homology at other sites in the genome. We are able to produce ZFNs that discriminate between these similar targets and still return genome-wide fidelity that is similar to or superior to any reported nuclease.

Our studies tested both four- and six-finger proteins, which allowed for an initial assessment of how target length influences activity, homologue discrimination and global specificity. Complex behaviour was observed. In the CCR5 studies, extra fingers substantially and consistently improved the activity of the right ZFNs (Fig. 4c, compare 46704 versus 46702 and 46705 versus 46703), but similar extensions to the left ZFNs yielded more modest effects (both increased and decreased activity). However, increasing the length of the left ZFNs did reduce off-target activity in our unbiased genome screen from 1.46 to 0.5% aggregate cleavage (Table 1). In our HBB studies, extension of the left ZFN—but not the right one—negatively impacted homologue discrimination. One possible explanation for this result is that under our study conditions the left ZFN plays a relatively more important role in distinguishing HBB from HBD, and that extension of the its target lessens the impact of binding the single mismatch that distinguishes the HBB and HBD sequences. In addition, here we report examples of nucleases that offer both high specificity and activity as well as examples where specificity is achieved at a small cost of activity. It is clear there is much left to be learned about how target length and composition influences the performance of ZFNs and other designed nucleases.

Finally, we have focused the MR-B1H system on zinc fingers because we reasoned the complex intra- and inter-finger interactions that lead to the context-specific activity of many zinc fingers may provide the sensitive ‘all or nothing' behaviour required to bind one target and discriminate against all similar sequences. Without context dependence, the modular TALE domain and RGEN systems seem likely to tolerate binding many similar targets. Nevertheless, the MR-B1H could be applied to these platforms as well. TALE domains expressed in the B1H system are functional (unpublished work) cas9-omega fusions have already proven functional in bacteria^62^. Therefore, it is feasible that libraries of TALE domains or cas9 could be expressed in the MR-B1H system to screen for versions of these proteins that offer the same levels of discrimination offered by the zinc fingers reported here. In sum, the MR-B1H system, applied to zinc fingers or the targeting domain of choice, has the potential to produce nucleases for other DNA target sequences with exclusive genome-wide fidelity.

## Methods

### B1H activity assay

Cells with desired omega-zinc finger and multi-reporter plasmid combinations were cultured until ‘cloudy' (OD600 ∼0.1 and above but not saturated) with rotation at 37 °C. Cells were pelleted and resuspended in non-selective minimal media (NM, see refs 42, 63 for all media recipes). Cells were expanded for 1 h at 37 °C. Cells were pelleted and washed four times in minimal NM that lacks histidine, uracil and isopropyl-β-D-thiogalactoside (IPTG). Cells were resuspended in 1 ml of this media, titred in 10-fold dilutions on rich plates with the appropriate antibiotics and stored at 4 °C overnight. On the basis of the overnight titre results, a similar number of cells harbouring various zinc-finger-binding site reporter combinations were titred in 10-fold dilutions on selective plates to provide side-by-side comparisons. These plates were grown for 24 h at either 30 or 37 °C.

### Growth rate test of liquid B1H assay

Cells with appropriate omega-zinc finger and multi-reporter plasmids were cultured overnight in supplemented minimal media to saturation with appropriate antibiotics but no selection or counter-selection pressure. Cells were diluted 1:150 into a optically clear flat bottom 96-well plate (Corning, 3635) with 150 μl of minimal media (NM), appropriate antibiotics, 3-aminotriazole and either no URA3 inhibitor, 6-azauracil (2 pg ml^−1^) or 5-fluoroorotic acid (2 mM) (Fermentas now ThermoScientific, R0812) per well. These represent no URA3 selection, positive URA3 selection and negative URA3 selection, respectively. The plate was sealed with optically clear breathable film (Sigma, Z380059) and placed in a plate reader. The plates were grown shaking at either 30 or 37 °C, double orbital (120 r.p.m.). OD600 measurements for each well were recorded every 10 min for 24 h and normalized to a blank. Doubling times in log phase were calculated.

### Fluorescence test of liquid B1H assay

Cells with appropriate omega-zinc finger and multi-reporter plasmids were cultured until ‘cloudy' (OD600 0.1 and above but not saturated). Cells were pelleted and resuspended in NM. Cells were expanded for 1 h at 37 °C. Cells were pelleted and washed four times in minimal media that lacks histidine, uracil and IPTG. Cells were resuspended in 1 ml of this media, titred in 10-fold dilutions on rich plates with the appropriate antibiotics, and stored at 4 °C overnight. On the basis of the overnight titre results, a volume of the culture held at 4 ^o^C that contains 10 million cells was used to start a 15-ml culture of minimal media containing 100 μM IPTG and various inhibitors as indicated (6-aza (2 pg ml^−1^), 5-FOA (2 mM), and/or 3-AT (5 mM)). These cultures were grown from 16–24 h but not allowed to reach OD600 above 0.8. Cells were recovered and resuspended in PBS plus 0.1% Tween. The mean fluorescence of each sample was measured with a BD LSRII Multi-Laser Analyzer with HTS (BD Biosciences, Sparks, MD, USA). Mean fluorescence values were determined from at least 20,000 cells. Each zinc-finger–reporter pair was assayed in triplicate.

### Pool assembly of zinc-finger libraries

In principle our four-finger zinc-finger libraries were assemble as described in prior works^38^,^41^,^52^,^54^. We used our previously selected pools of individual zinc fingers^41^ as PCR templates to build four-finger libraries guided by the desired 12-nt target. Therefore, for each 12-nt target a new four-finger ‘pool library' was assembled. To create this library, individual pools that corresponding to each 3-nt subsite of the 12-nt target were used as the templates for PCR (Supplementary Fig. 4). For example, for the original right CCR5 target, 5′-AAA-CTG-CAA-AAG-3′, the AAA, CTG, CAA and AAG pools were used as the PCR templates for each finger of the library. PCR primers were designed to provide overlap so that these PCR pools could be assembled in a second round of PCR by overlapping PCR in the order N terminus-pool^AAG^-pool^CAA^-pool^CTG^-pool^AAA^-C terminus (zinc fingers bind DNA anti-parallel to the 5′–3′ sequence of DNA). The 5′- and 3′-most oligonucleotides code for KpnI and XbaI restriction sites, respectively. Digestion of the final, four-finger PCR pool assembly with these two restriction enzymes allows cloning, in frame, into our expression vectors at the 3′-end of the omega coding sequence.

PCR reactions were carried out according to the manufacturers' guidelines using Expand High Fidelity Plus (Roche, 04 743 733 001). For each individual zinc-finger pool, 8, 15- to 20-cycle 50-μl PCR reactions were performed. The PCR products were recovered by gel purification and used as the template for the assembly rounds of PCR, again using Expand High Fidelity Plus. The final four-finger pool assemblies were recovered by gel purification and used as the template for a final library expansion that only includes the 5′- and 3′-most oligonucleotides and 30 cycles of PCR. This final expansion was recovered by PCR purification (Qiagen) and digested with KpnI and XbaI according the manufacturer's guidelines (NEB). The digested product was recovered by gel purification (Qiagen Minelute) and eluted in a small volume of buffer (typically 10 μl or less) to maintain high concentration. Finally, 20- to 100-μl ligations into the expression vectors were performed using T4 DNA Ligase (NEB). In each ligation digested vector is present at a concentration of 1 μg per 10 μl of ligation. Digested library insert is added to a final concentration that provides a 5 × insert to vector molar ratio. For most of our libraries this is ∼500 ng of insert per 1 μg of vector. Ligations were incubated at 16 °C overnight (minimum of 16 h). The ligation was ethanol precipitated and resuspended in 1 μl of water per 1 μg of vector backbone used in the ligation. Detailed zinc-finger and vector sequences are provided in Supplementary Notes 2–4.

### Zinc-finger protein selections

To select four-finger proteins able to bind 12-nt targets of interest, the library assemblies described above were paired with the appropriate multi-reporter vector and transformed into our selection strain. Detailed binding site and vector sequences are provided in Supplementary Notes 2–5. For each transformation 1 μl of the ligation (loosely representing 1 μg of library vector) was paired with 1 μl of multi-reporter vector (500 ng–1 μg) and transformed into our Δ*rpoZ* selection strain by electroporation. In this way the library build is being recovered and assayed in one step. Typically, 1 μl of library will give us 5 × 10^7^ transformants in our selection strain. Therefore, to assay well over 10^8^ library members a standard selection would include four or five transformations. After electroporation, the cells were expanded in rich media (SOC, consisting of Difco SOB cat# 244310, with 0.5% glucose) for 1 h at 37 °C. The cells were pelleted and resuspended in 10 ml of NM that contained kanamycin and ampicillin. The cells were again expanded for 1 h at 37 °C. The cells were pelleted and washed four times in NM without uracil or histidine. The cells were resuspended in 1 ml of NM without uracil and histidine. 20 μl of this resuspension was titred in 10-fold dilutions on rich media plates while the remaining 980 μl stored at 4 °C overnight.

The following day, cell titres provide a cell count per volume. A total of 2 × 10^8^ cells were plated on NM plates containing 5 mM 3-AT to provide a low-stringency positive selection and remove non-functional zinc fingers. These plates were incubated at 37 °C for 36–48 h. In all cases reported here at least 10,000 cells survived this low-stringency selection. After incubation, cells were collected, DNA recovered and precipitated. This DNA was transformed again with the appropriate multi-reporter plasmid. Again, after electroporation the cells were expanded in rich media (SOC) for 1 h at 37 °C. The cells were pelleted and resuspended in 10 ml of non-selective minimal media (NM) that contained kanamycin and ampicillin. The cells were again expanded for 1 h at 37 °C. The cells were pelleted and washed four times in NM without uracil or histidine. The cells were resuspended in 1 ml of NM without uracil and histidine. A volume of 20 μl of this resuspension was titred in 10-fold dilutions on rich media plates while the remaining 980 μl stored at 4 °C overnight. On the basis of the overnight titre results, a volume that contains 1x10^7^ cells was used to start a 15-ml culture of minimal media containing 100 μM IPTG and 10 mM 3-AT and 2 mM 5-FOA. These cultures were grown for 24–30 h at 30 °C but not allowed to reach OD600 above 0.8. Cells were recovered and resuspended in PBS plus 0.1% Tween for sort preparation.

### Fluorescence-activated cell sorting and recover

For each experiment a negative control was grown in minimal but non-selective media. This control did not express a DNA-binding domain (DBD) able to activate either reporter and could thus be used to establish background levels of GFP and mCherry expression using the exact reporter vectors and bacterial strain as the experimental samples. These levels were used as limits for cell gating and classification of GFP- and/or mCherry-positive cells (see Fig. 2c as an example). Therefore, desired experimental samples prepared as above were sorted directly into rich media (SOC) using a BD FACSVantage SE w/DiVa instrument (BD Biosciences) at 16 psi with a 70-μm nozzle using sterile PBS as sheath fluid. Cells were characterized using forward and side scatter parameters and GFP and mCherry fluorescent proteins were excited via 488 and 568 nm laser lines, respectively. Emitted fluorescence was collected using a 530/30 bandpass filter for GFP and a 600 longpass filter for mCherry. Data were acquired and analysed using FACSDiVa software (BD Biosciences). In all, 30,000 events were collected for each sorted population. A volume of the recovered events were plated on rich media to recover 250–500 colonies of bacteria and grown overnight at 37 °C. From 24–48 individual colonies, the zinc-finger coding sequences were amplified by PCR and sequenced for each target selection. Coding sequences were translated enriched amino acids compared for analysis.

### ZFN construct for cellular studies

ZFN protein sequences and expression constructs used for cellular studies are provided in Supplementary Note 6. Unless otherwise noted all constructs were generated using standard molecular biology methods.

### SELEX studies

An oligonucleotide target library was synthesized bearing the sequence: 5′-CAGGGATCCATGCACTGTACGCCCNNNNNNNNNNNNNNNNNNNNNNGGGCCACTTGACTGCG GATCCTGG-3′ where ‘N' denotes a mixture of all four bases. The library was converted to double-stranded duplex by annealing 2 nmol of library oligo with 6 nmol of 3′ library primer (5′-CCAGGATCCGCAGTCAAGTGG-3′) in 100 μl 1 × PCR Master (Roche) supplemented to 1.2 mM of each dNTP and 5 mM MgSO_4_, followed by incubation at 95 °C for 2 min, 94 °C for 5 min, 58 °C for 5 min and 72 °C for 15 min.

For the first assay cycle, ZFNs were expressed directly from plasmid templates using a TnT-coupled transcription–translation system (Promega) and the manufacturer's recommended conditions with buffers supplemented to 10 mM ZnCl_2_. Expressed ZFNs contained a triple Flag tag fused to their N terminus. A volume of 12 μl of TnT reaction mix was then mixed with 200 pmol of library duplex in a total volume of 100 μl of binding buffer (5 mM dithiothreitol, 10 μM ZnCl_2_, 5 mM MgCl_2_, 0.01% BSA Fraction V and 100 mM NaCl in PBS (calcium free)). After incubation for 50 min protein–DNA complexes were captured on anti-FLAG M2 magnetic beads (Sigma) and washed five times with wash buffer (5 mM dithiothreitol, 10 μM ZnCl_2_, 5 mM MgCl_2_, 0.01% BSA Fraction V and 100 mM NaCl in PBS (calcium free)). Bound target was PCR amplified using the 3′ library primer (above) and a 5′ library primer (5′-CAGGGATCCATGCACTGTACG-3′), and the resulting amplicon was used as input for additional cycles of enrichment. Protein expression and binding conditions for these subsequent cycles were identical to the conditions used in the first round. After three cycles, recovered DNA fragments were sequenced using an Illumina MiSeq system. The protocol for adding the Illumina sequencing primers and sequencing is as described in reference 6464.

SELEX FASTQ sequences from the MiSeq were adapter trimmed using SeqPrep (J. St. John, unpublished, https://github.com/jstjohn/SeqPrep). SELEX library sequences were further filtered by custom python scripts for correct length and fixed flanking region composition (exact match). In all, 200 randomly sampled filtered sequences were used as input to the GADEM motif discovery programme with options maskR=0 fullscan=0 gen=3. Position frequency matrices discovered by GADEM^65^ were then aligned to the intended sequence and reverse complemented if necessary. Matrices longer than the intended sequence were trimmed to only those regions overlapping the intended sequence according to the highest-scoring alignment, yielding the final matrices provided in Fig. 3.

### Gene modification of endogenous CCR5 and CCR2

To screen ZFN pairs for NHEJ-mediated gene modification, K562 cells were cultured in RPMI 1640 media (Invitrogen) supplemented with 10% (v/v) FBS, 2 mM L-glutamine, 100 U ml^−1^ penicillin and 100 mg ml^−1^ streptomycin. Cells (1–2 × 10^5^) were nucleofected with expression plasmids (400 ng each) using the Amaxa 96-well shuttle system (Amaxa Biosystems/Lonza) according to manufacturers' instructions (setting 96-FF-120). Cells were collected 3 days post-transfection and genomic DNA was extracted using the QuickExtract DNA Extraction Solution (Epicentre Biotechnologies) according to the suppliers' instructions. Frequency of gene modification by NHEJ was evaluated by deep sequencing using an Illumina MiSeq. Primers used for the primary PCR were the following: CCR2 out PCR primer: CCR2Out4LZF, 5′-CTGTCCACATCTCGTTCTCG-3′; CCR2Out741LZR, 5′-GGTGAAGATGACTCTCACTG-3′. CCR2 miSeq PCR primer: CCR2MS134LZF, 5′-ACACGACGCTCTTCCGATCTnnnnTGCCTCCGCTCTACTCGC-3′; CCR2MS294LZR, 5′-GACGTGTGCTCTTCCGATCTCCACAATGGGAGAGTAATAAGAA-3′. CCR5 out PCR primer: R5-cel1-F1, 5′-AAGATGGATTATCAAGTGTCAAGTCC-3′; R5-cel1-R1, 5′-CAAAGTCCCACTGGGCG-3′. CCR5 miSeq PCR primer:

R5-As-miseq-Fn, 5′-ACACTCTTTCCCTACACGACGCTCTTCCGATCTnnnnnGCCAGGTTGAGCAGGTAGATG-3′;

R5-As-R-mp, 5′-AGACGTGTGCTCTTCCGATCTGCTCTACTCACTGGTGTTCATCTTT-3′.

### Capture assay

To capture IDLV at sites of ZFN cleavage, K562 cells (ATCC CCL-243) were cultured in RPMI medium supplied with 10% FBS. One day before ZFN transfection, cells (1.5 × 10^5^) were infected with IDLV at a multiplicity of infection of 100. Approximately 20 h later, cells (2 × 10^5^) were nucleofected (Lonza 96-well shuttle system, Nucleofector SF Solution and Program 96-FF-120) with each pair of ZFN-expressing plasmids. Nucleofections were performed in triplicate, using 200 ng of each plasmid, for CCR5-targeted ZFNs, and in quadruplicate, using either 400 or 800 ng of each plasmid for HBB-targeted ZFNs. After 1 day cultures were transferred to a six-well plate.

On day 14 and day 28 post-transfection, genomic DNA was isolated (Qiagen DNeasy Blood & Tissue Kit) and processed to isolate insert-genome junctions as described^66^ (steps 1–38), except for the use of an 8-s extension time, and annealing temperatures of 53, 47 and 50 °C for each amplification step. Candidate products were then processed for high throughput sequencing via MiSeq using standard methods.

DNA sequence reads were then processed as follows: first, nonidentical reads were filtered for correct priming and adapter sequences, and the resulting sequences mapped to the genome. Next, junction coordinates were mapped and hits within 1 kb of each other were merged into clusters while keeping count of integration events. A 1-kb cutoff was used. Next, to reduce background signal from capture into random, cell cycle, or environmentally induced DSBs, clusters were filtered to contain integrations from at least two out of three replicates of ZFN-treated samples and at most one out of three replicates of control were scored as potential targets. These clusters were ranked by the total number of unique integrations in the ZFN-treated samples.

### Production of integrase-defective lentiviral vectors

Recombinant IDLVs were generated from the HIV-derived self-inactivating construct^67^ RRL–CMV–VENUS, which expresses the yellow fluorescent protein (YFP) Venus protein under control of the CMV promoter. The integrase-defective packaging plasmid pMDLg/pRRE containing the D64V point mutation^68^ was used to generate IDLV stocks as previously described^69^. Their titres were determined by transduction of 293 T cells analysed for YFP VENUS expression by flow cytometry.

### Off-target analysis

For the off-target analysis, K562 cells were transfected with ZFN-expressing plasmids and cultured essentially as described above in the section ‘Gene modification of endogenous *CCR5*, *CCR2*'. Amplicons from candidate off-target loci were then amplified using the PCR primers shown in Supplementary Data 1 (designed using Primer3 with the following optimal conditions: amplicon size of 200 nucleotides, a Tm of 60 °C, primer length of 20 nucleotides and GC content of 50%). Adapters were added for a second PCR reaction to add the Illumina library sequences (ACACGACGCTCTTCCGATCT forward primer and GACGTGTGCTCTTCCGAT reverse primer), followed by MiSeq sequencing using standard methods. For off-targets where evidence of indels were found, the putative binding sites for each set of zinc fingers are shown in Supplementary Table 1.

In detail, genomic DNA was purified with the Qiagen DNeasy Blood and Tissue Kit (Qiagen). Regions of interest were amplified in 50 μl using 250 ng of genomic DNA with Phusion (NEB) in Buffer GC with 200 μM dNTPs. Primers were used at a final concentration of 0.5 μM and the following cycling conditions: initial melt of 98 °C for 30 s, followed by 30 cycles of 98 °C for 10 s, 60 °C for 30 s and 72 °C for 15 s, followed by a final extension 72 °C for 10 min. PCR products were diluted 1:200 in H_2_O. A volume of 1 μl diluted PCR product was used in a 10-μl PCR reaction to add the Illumina library sequences with Phusion (NEB) in Buffer GC with 200 μM dNTPs. Primers were used at a final concentration of 0.5 μM and the following conditions: initial melt of 98 °C for 30 s, followed by 12 cycles of 98 °C for 10 s, 60 °C for 30 s, 72 °C for 15 s, followed by a final extension 72 °C for 10 min. PCR products were pooled and purified using the Qiagen Qiaquick PCR Purification Kit (Qiagen). Samples were quantitated with the Qubit dsDNA HS Assay Kit (Life Technologies). Samples were diluted to 2 nM and sequenced on an Illumina MiSeq Instrument (Illumina) with a 300 cycle sequencing kit. To compare indels across samples and controls, the statistical test described in ref. 21^21^ was applied to the number of sequences scored as indels and the total number of sequences for both the ZFN-treated sample and the cognate control. *P* values were then adjusted for multiple comparisons with a Bonferroni correction.

## Additional information

**How to cite this article:** Oakes, B. L. *et al.* Multi-reporter selection for the design of active and more specific zinc-finger nucleases for genome editing. *Nat. Commun.* 7:10194 doi: 10.1038/ncomms10194 (2016).

## Supplementary Material

Supplementary Information Supplementary Figures 1-9, Supplementary Table 1 and Supplementary Notes 1-6

Supplementary Data 1 PCR primers to amplify potential genomic nuclease targets

## Figures and Tables

**Table 1:**
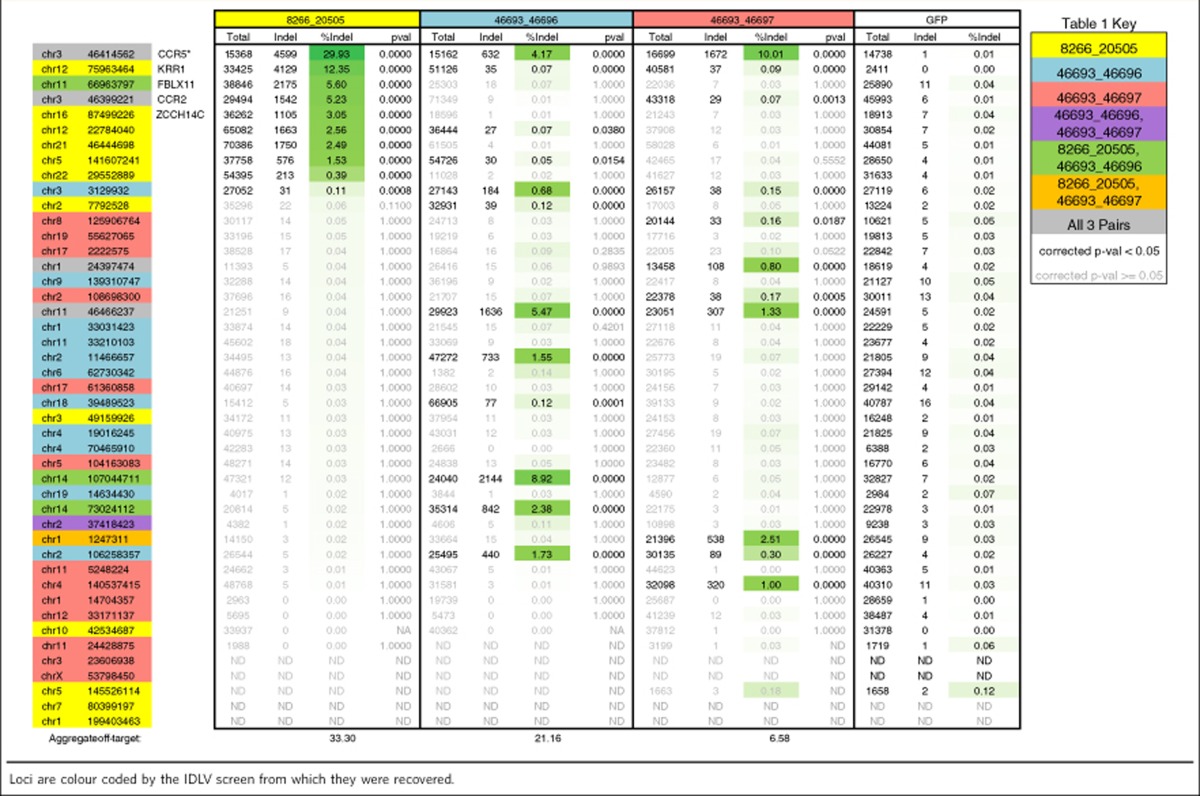
Indel frequencies recovered at genomic loci as predicted across IDLV screens using four-fingered zinc-finger monomers.

**Table 2:**
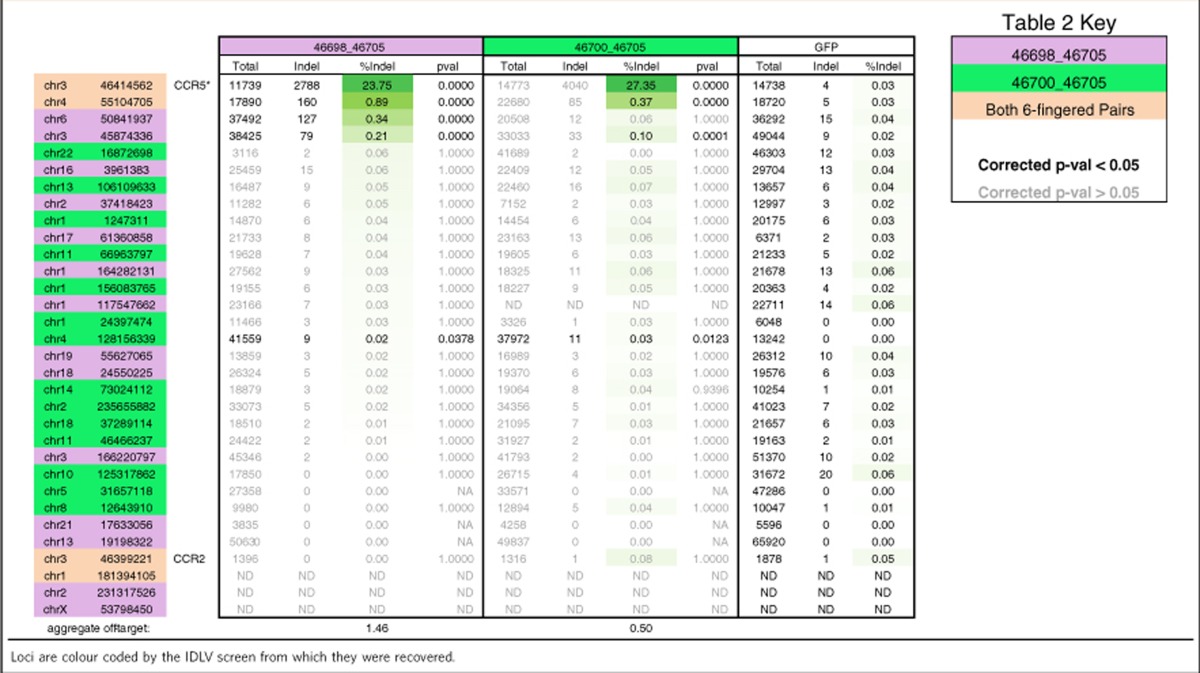
Indel frequencies recovered at genomic loci as predicted across IDLV screens using six-fingered zinc-finger monomers.
