# Supplementary material for: Multi-reporter selection for the design of active and more specific zinc-finger nucleases for genome editing
Source: Nat Commun. 2016 Jan 7;7:10194. doi: 10.1038/ncomms10194 (PMC4729830; doi:10.1038/ncomms10194)
Supplement: Supplementary Information — Supplementary Figures 1-9, Supplementary Table 1 and Supplementary Notes 1-6 [file ncomms10194-s1.pdf]

Supplementary Figures

Supplementary Figure 1

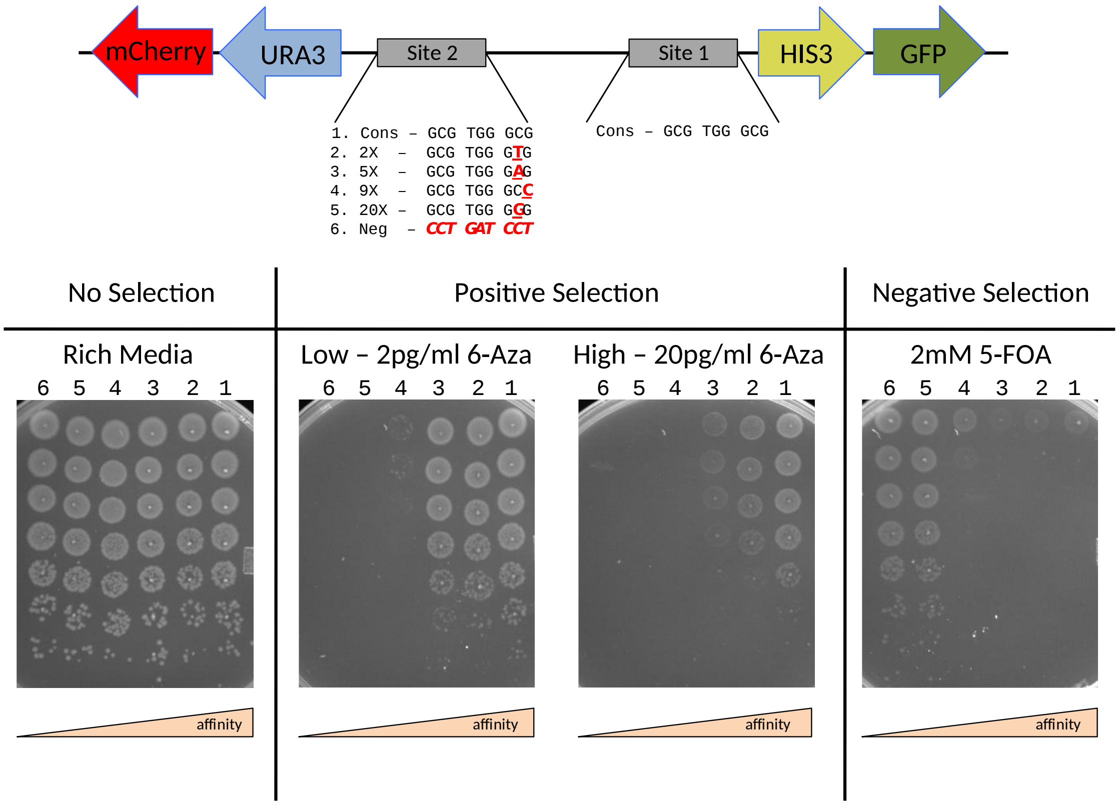

Supplementary Figure 1. – Plate titrations with site 1 fixed and site 2 varied, focused on URA3 variability, rather than HIS3 variability in the main Fig. 1. In each case, omega-zif268 is expressed and paired with a multi-reporter vector that has the consensus zif268 target driving HIS3/GFP and one of sequences 1-6 driving URA3/mCherry. URA3 provides the advantage that one can select for its activation (competitive inhibitor 6-azauracil) or against its activation (suicide inhibitor 5-Fluoroorotic acid). Cells were grown in liquid culture, washed in minimal media, and titered in 10-fold dilutions on the plates above. On rich media (left), all cultures grow out to the 7th dilution. However, with a positive selection (6-aza, middle) only cells that contain a high affinity binding site to drive URA3 survive the conditions. Conversely, when selecting against URA3 activation (right), only the weakest of binding sites survive the selection.

Supplementary Figure 2

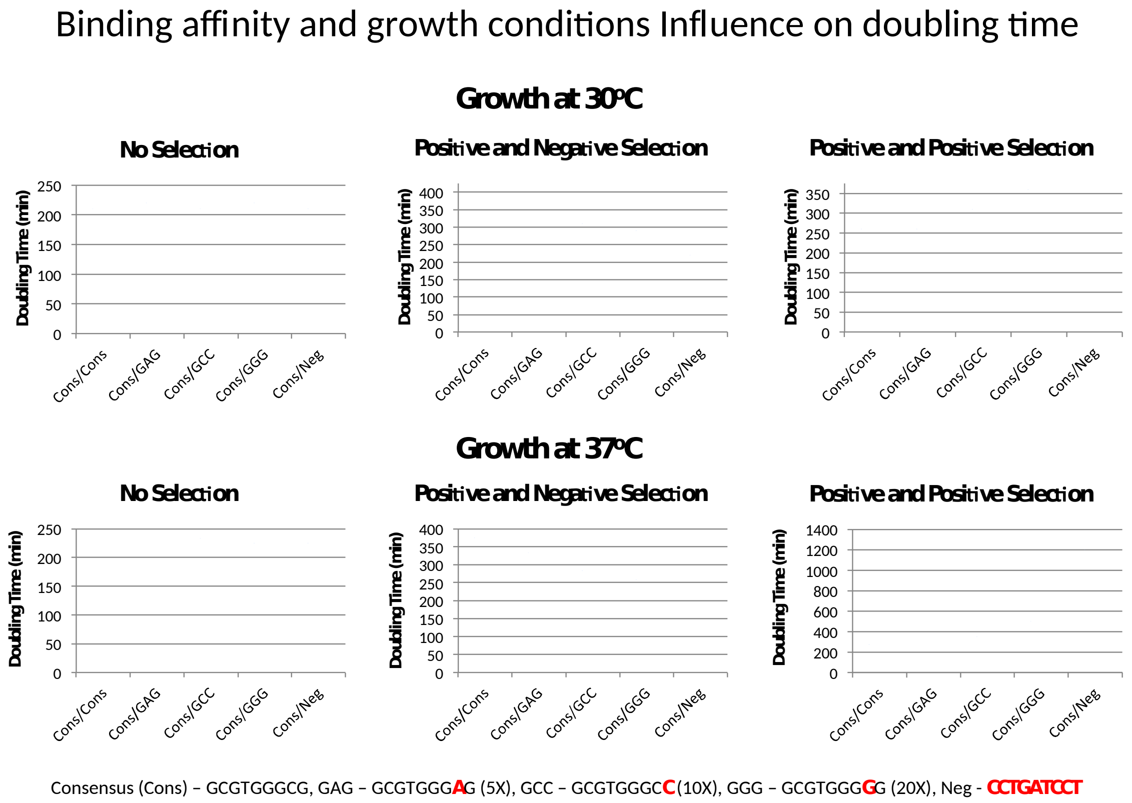

Supplementary Figure 2. – Zif-268 and binding site growth rates. As in supplementary figure 1, omega-zif268 was expressed in combination with its consensus target sequence driving HIS3/GFP and one of a suite of binding sites driving URA3/mCherry. Cells were then grown in liquid cultures, as noted in the histograms above, either without selection, or with selection for HIS3 activation coupled with a negative (middle) or positive (right) selection of URA3. These tests were done at both 30 ℃ (top) and 37ºC (bottom). Doubling times are clearly related to the affinity of the interactions that drive URA3 and the selection conditions.

Supplementary Figure 3

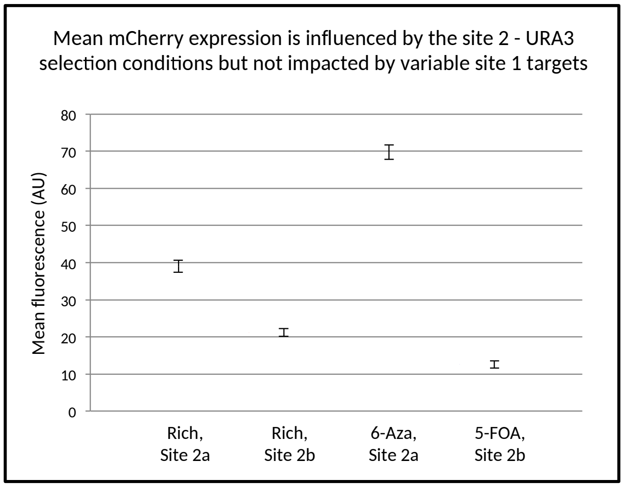

Supplementary Figure 3. –Fluorescence is not influenced by the interactions that drives the alternative reporter. Complementary to main figure 1D, cells that express omega-zif268 were grown with a fixed binding site to drive URA3/mCherry (either the zif268 consensus – site 2a, or a negative control – site 2b) paired with a suite binding sites that drive HIS3/GFP. Cells were then grown in either rich media or media that selects for or against URA3 activation. While mCherry output is clearly related to the growth conditions and the sequence at site 2, each group is consistent indicating that the variable affinity that drives HIS3/GFP expression (see main figure 1D) does not influence the activation of URA3/mCherry. Standard error of the mean is shown, 3 experiment replicates were measure for each group.

Supplementary Figure 4

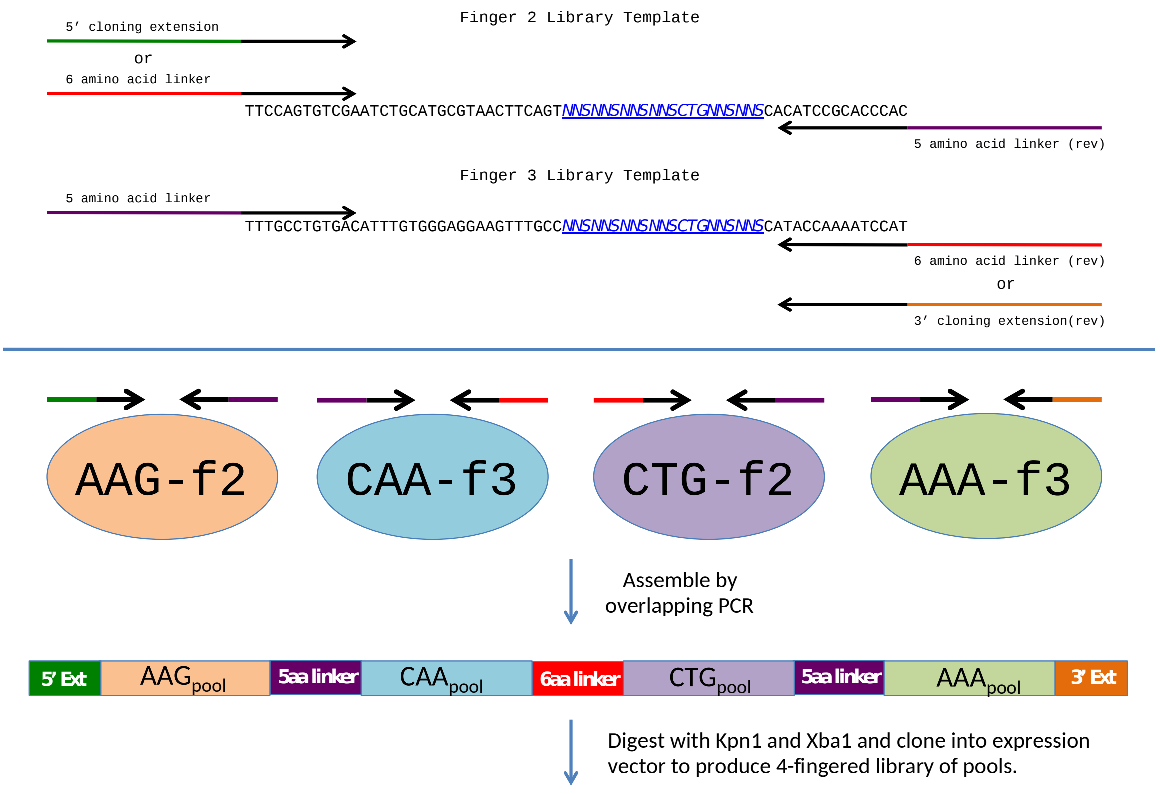

Supplementary Figure 4. Zinc finger pool assembly. Zinc fingers were amplified from previously selected pools for each of the 64 possible 3-nt targets with both zif268 finger 2 and finger 3 libraries. Four-fingered libraries were constructed by alternating finger 2 and finger 3 pool amplifications that would correspond to the target of interest. Using the CCR5 right target as an example (AAA-CTG-CAA-AAG), PCR primers were designed to amplify each finger pool (top) in such a way as to provide overlapping linker sequences (color coded as 5 amino acid:purple and 6 amino acid: red) that would allow assembly in the order N-terminus-AAG_{f2pool} – CAA_{f3pool} – CTG_{f2pool} – AAA_{f3pool}-C-terminus. In a second round of PCR, the AAG_{f2pool} – CAA_{f3pool} and CTG_{f2pool} – AAA_{f3pool} pairs are assembled using the designed linker overlap. In the final assembly, the middle, 6 amino acid linker overlap is used to assemble the full-length four–finger pool library. A final round of PCR is used to expand this assembly. DNA is recovered, digested with restriction enzymes complementary to sites installed in the 5’ and 3’ extension primers (KpnI and XbaI) and ligated into the omega expression vector.

Supplementary Figure 5

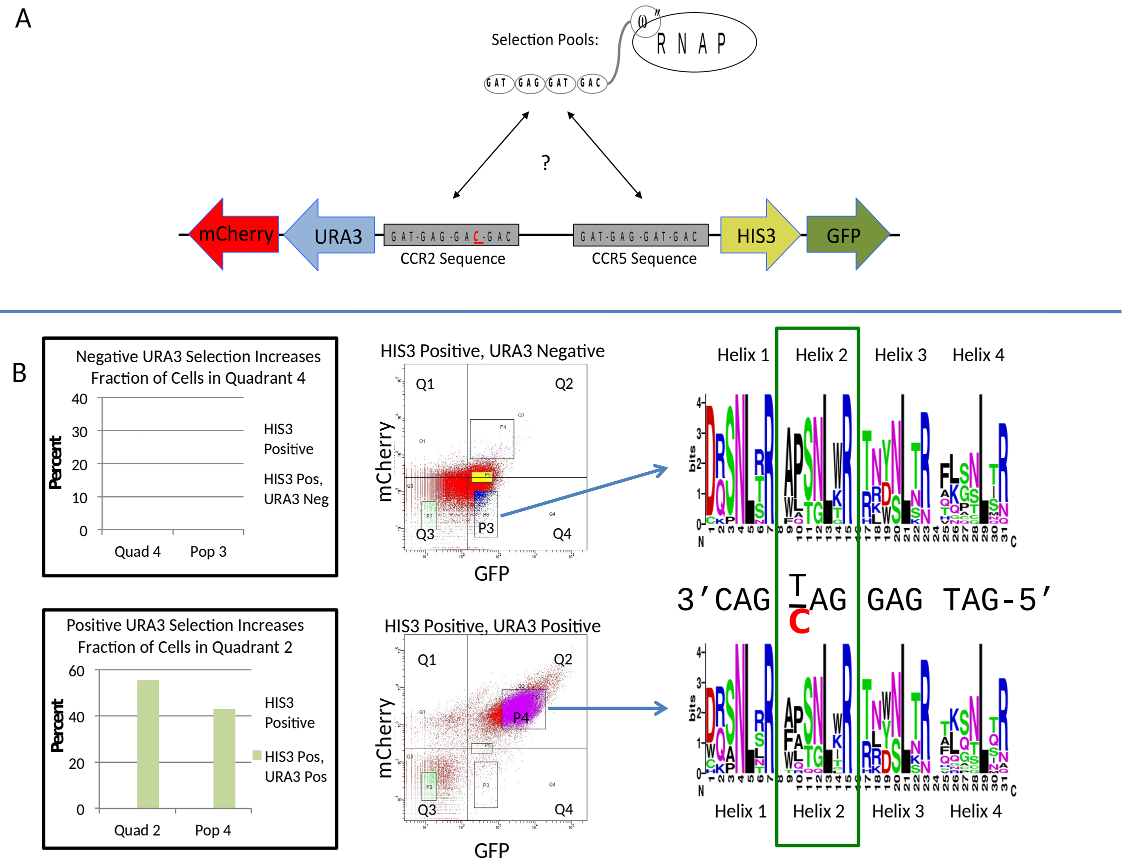

Supplementary Figure 5. Selection of zinc fingers that discriminate between the similar LEFT CCR5 and CCR2 targets. **A.** Complementary to main Figure 2, the CCR5 LEFT target is placed in front of the promoters that drive HIS3 expression, with the corresponding CCR2 sequence in front of URA3. Zinc finger pools previously selected to bind each 3bp sub-site of the desired target are used as PCR templates to assemble a 4-fingered library, illustrated as rainbow-colored ovals. To select 4-fingered members of this library that are able to discriminate between the desired targets, cells can be grown under conditions that are inhibited by URA3 expression but require HIS3 activation. **B.** Selection conditions influence the enriched amino acids that correspond to the target mismatch. Using the library described in A, selection for HIS3 activation but against URA3 activation increases the fraction of the population in the GFP positive, mCherry negative quadrant 4 in comparison to a HIS3 positive selection alone (*Top*). Using the same library, selection for both HIS3 and URA3 activation increases the fraction of the population in the GFP positive, mCherry positive quadrant 2 in comparison to a HIS3 positive selection alone (*Bottom*). Sequencing the zinc fingers recovered from stringent populations of these selection conditions allows a comparison of the amino acids enriched in the helix that corresponds to the difference in the desired and counter target (*green box*).

Supplementary Figure 6

CCR5 Targets and their relation to CCR2

Original Target:

```
CCR5    …caacatgctgGTCATCCTCATCCtgatAAACTGCAAAAGgctgaagagcatgactgacat…
CCR2    …caacatgctgGTCGTCCTCATCTtaatAAACTGCAAAAAgctgaagtgcttgactgacat…
```

Shift Target:

```
CCR5    …caacatgctGGTCATCCTCATCCTGATaaactgCAAAAGGCTGAAGAGCATgactgacat…
CCR2    …caacatgctGGTCGTCCTCATCTTAATaaactgCAAAAAGCTGAAGTGCTTgactgacat…
```

```
        Left Targets:                         Right Targets

LEFT A:   ATC AGG ATG AGG             RIGHT A:   CAA AAG GCT GAA

LEFT B:           ATG AGG ATG ACC     RIGHT B:           GCT GAA GAG CAT
```

Supplementary Figure 6. A comparison of the original and shifted CCR5 targets in relation to the homologous CCR2 sequence. *Top*, the original CCR5 targets are bold and underlined with the homologous CCR2 sequence shown below. Mismatches between CCR5 and CCR2 are shown as red letters. The aligned, shifted target is shown below. The center of this target is only 6-nt 3' to the original target, however, by shifting and extending to an 18-nt target per monomer (similar to common TALEN architectures) we are able to pick up three CCR5:CCR2 mismatches per monomer binding site (red letters). *Bottom*, the shifted targets for the overlapping four-fingered libraries are shown with CCR2 mismatch positions shown in red.

Supplementary Figure 7

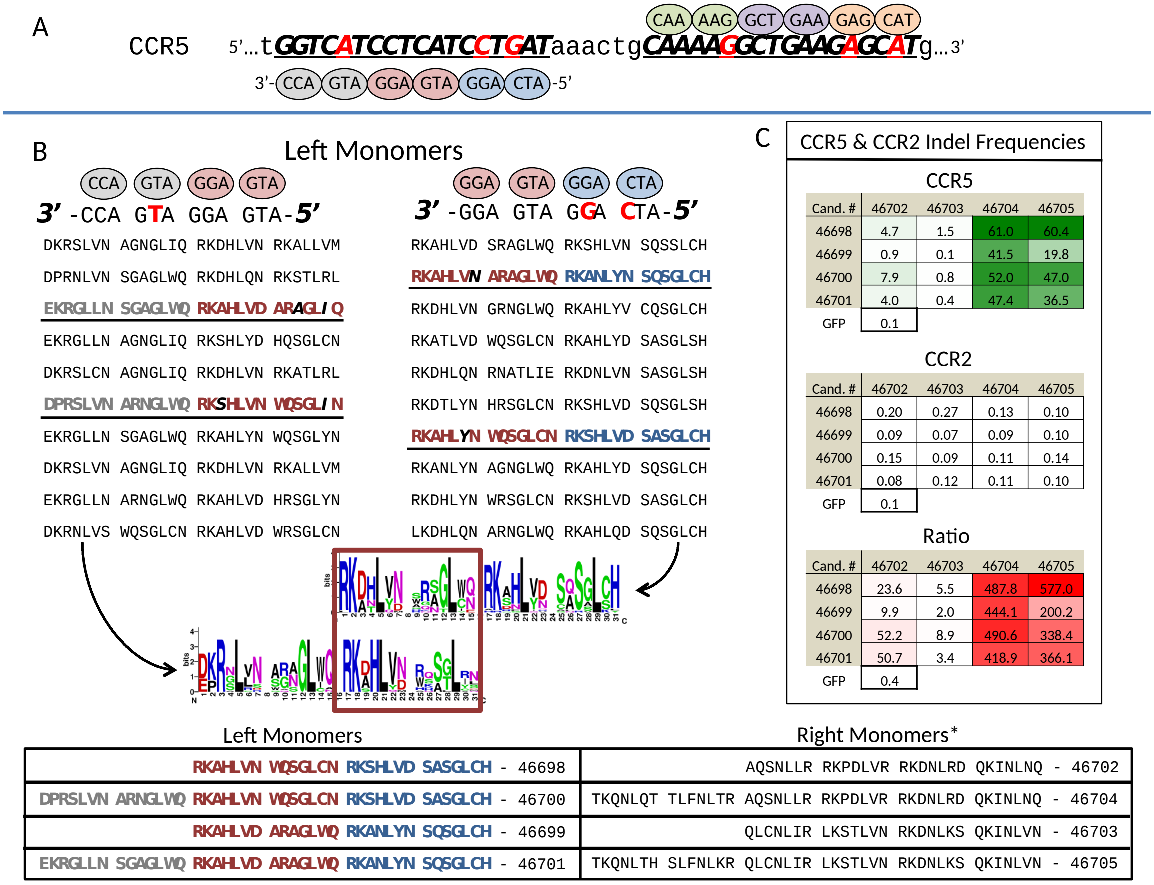

Supplementary Figure 7. Extending zinc finger targets to increase CCR2 discrimination; a complementary **"left target"** figure to Fig. 4 of the main text. **A.** The CCR5 target is shifted 6-nt 3' relative to the target in Figure 3 (bold and underlined). Mismatches with the CCR2 sequence are shown as red letters. Each ZFN monomer is increased from 4 to 6 fingers. **B.** For each target, two overlapping 4-fingered libraries are produced. The left monomer pools are shown here while the right monomer pools are shown in Fig. 4. Pools of these libraries are color coded to emphasize that the overlapping zinc fingers target the same sequences. Zinc fingers are selected from each pool by selection for the CCR5 sequence but against the CCR2 sequence. Targets are shown 3' to 5' to emphasize the overlap in the targets of the 4-fingered selections. From each of these selections, 10 of the selected ZFPs are shown. Candidates used to design the 4 and 6-finger monomers employed as nucleases are bold and underlined. All enriched amino acids for each of the 4-fingered selections are shown below as a sequence logo with the overlapping 2 fingers boxed in purple. **C.** Candidate ZFN pairs were expressed *in vivo* and the percentage of indels at CCR5 and CCR2 measured. Indel frequencies recovered at either target from cells that did not express a nuclease (GFP) are shown below each table. The ratio of CCR5 to

CCR2 indel frequency is shown below. **Table**. A table of the 4 and 6-fingered ZF helices used in the nuclease studies, shown N-term to C-term, is provided.

Supplementary Figure 8

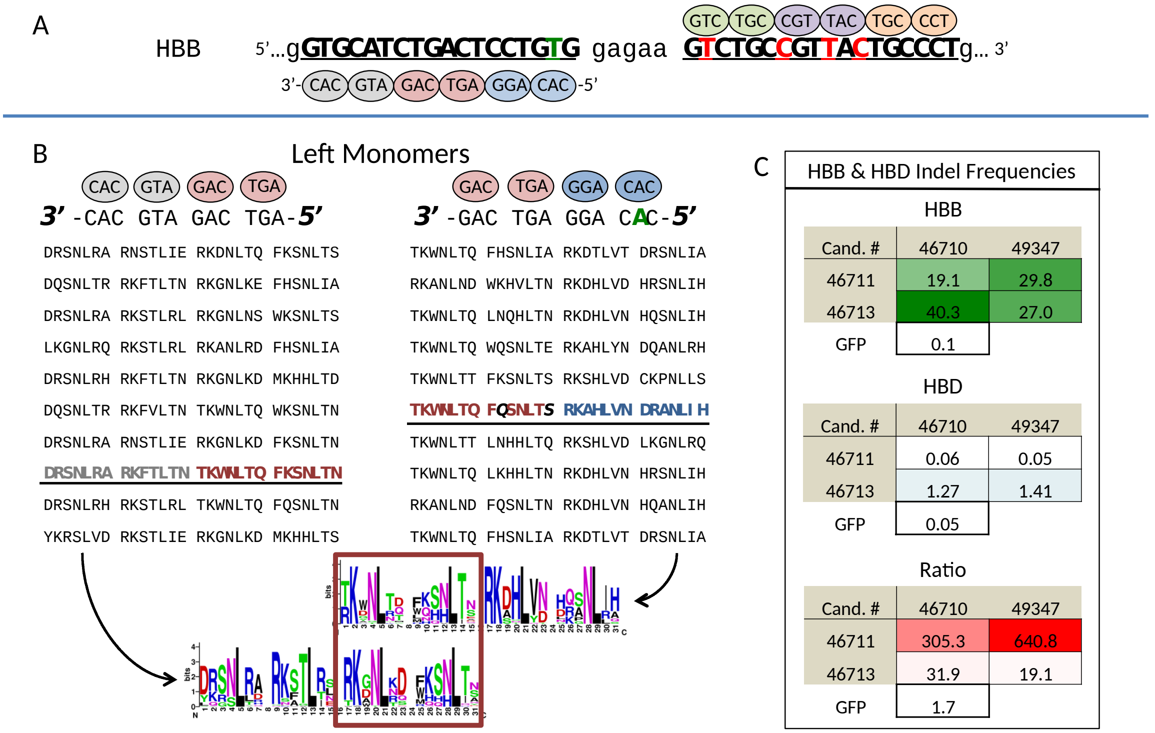

Left Monomers	Right Monomers*
TKWNLTQ FKSNLTN RKAHLVN DRANLIH - 46711	LKGNLLK MKHHLTE WPNSLKY DRSALIR - 46710
DRSNLRA RKFTLTN TKWNLTQ FKSNLTN RKAHLVN DRANLIH - 46713	RKQCLQR WPNSLKA DRSNLTK HKHHLTE WPNSLKY DRSALIR - 46347

Supplementary Figure 8. MR-B1H produced zinc fingers provide high HBB activity with strong discrimination against HBD; a complementary **"left target"** figure to Fig. 5 of the main text. **A**. The HBB target is shown, mismatches to the HBD sequence are shown as red letters. The sickle cell causing mutation that separates the left target here from the HBD sequence is shown as a green letter. **B.** For each target, two overlapping 4-fingered libraries are produced. The left monomer pools are shown here while the left monomer pools are shown in Fig. 5. Pools of these libraries are color coded to emphasize that the overlapping zinc fingers target the same sequences. Zinc fingers are selected from each pool by selection for the HBB sequence but against the HBD sequence. Targets are shown 3' to 5' to emphasize the overlap in the targets of the 4-fingered selections. From each of these selections, 10 of the selected ZFPs are shown. Candidates used to design the 4 and 6-finger monomers employed as nucleases are bold and underlined. All enriched amino acids for each of the 4-fingered selections are shown below as a sequence logo with the overlapping 2 fingers boxed in purple. **C.** Candidate ZFN pairs were expressed *in vivo* and the percentage of indels at HBB and HBD measured. Indel frequencies recovered at

either target from cells that did not express a nuclease (GFP) are shown below each table. The ratio of HBB to HBD indel frequency is shown below. **Table**. A table of the 4 and 6-fingered ZF helices used in the nuclease studies, shown N-term to C-term, is provided.

Supplementary Figure 9

ZFN 46698

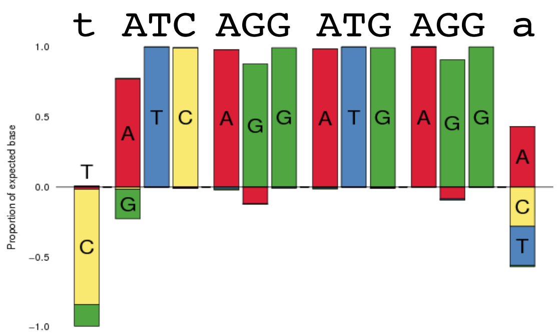

ZFN 46700

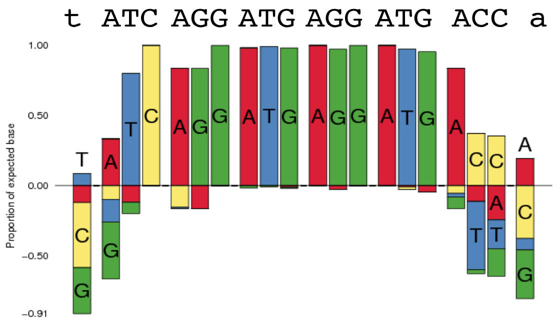

ZFN 46705

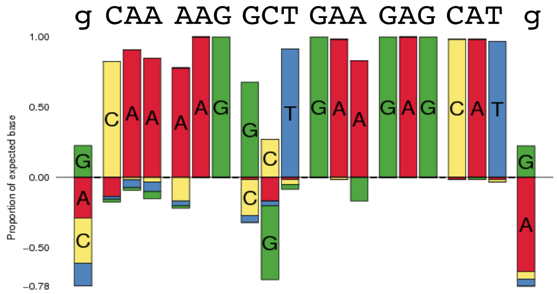

Supplementary Figure 9. SELEX results for the extended ZFPs used to target the shifted CCR5 sequence. The SELEX binding specificities for zinc fingers used in the IDLV assay to cleave the shifted CCR5 sequence shown here were produced as detailed in the method. Results for the zinc fingers engineered to bind the shifted CCR5 target are shown here while SELEX results for the original CCR5 target are shown in Fig. 3

Supplementary Table 1

Supplementary Table 1

coordinates		locus	gap (bp)	8266 site	20505 site
chr3	46414562	CCR5*	5	GATGAGGATGAC	AAACTGCAAAAG
chr12	75963464	KRR1	5	GATGAGGAGGCC	AAACTGGAAATG
chr11	66963797	FBLX11	5	GATGAGGTCTCA	AAACTGTAATAG
chr3	46399221	CCR2	5	GATGAGGACGAC	AAACTGCAAAAA
chr16	87499226	ZCCH14C	5	GAGGAGGCCTCT	AAACTGTAACAG
chr12	22784040		5	GATGAGTTAGAA	AAACTGTAATGG
chr21	46444698		6	GATGAGCATTCA	AGACTGGATAAG
chr5	141607241		5	GATGAGGAACAG	AAACTGGAAGTA
chr22	29552889		5	GATGAGGAAATT	AAACTGGAATAG
chr3	3129932		6	GCAGAGGAGGTG	CCACTGCAAAAG

				46693 site	46696 site
chr3	46414562	CCR5*	5	GATGAGGATGAC	AAACTGCAAAAG
chr12	75963464	KRR1	5	GATGAGGAGGCC	AAACTGGAAATG
chr12	22784040		5	GATGAGTTAGAA	AAACTGTAATGG
chr5	141607241		5	GATGAGGAACAG	AAACTGGAAGTA
chr3	3129932		6	GCAGAGGAGGTG	CCACTGCAAAAG
chr2	7792528		ND	ND	ND
chr11	46466237		6	GAGGAGGAAGAC	TCACTGCAAAAG
chr2	11466657		6	GAGGAGGAGGCA	GCAAAACAAAGC
chr18	39489523		5	GATGAGGATCCA	GGCAATGAAAAG
chr14	107044711		6	GATGAGGAGGTG	ACATTGCAAGAG
chr14	73024112		6	GAGAAGGAGCAC	GCAAAGCAAAAG
chr2	106258357		6	GAAGAGGAGTAC	GAGCTGCAAAGC

				46693 site	46697 site
chr3	46414562	CCR5*	5	GATGAGGATGAC	AAACTGCAAAAG
chr12	75963464	KRR1	5	GATGAGGAGGCC	AAACTGGAAATG
chr3	46399221	CCR2	5	GATGAGGACGAC	AAACTGCAAAAA
chr3	3129932		6	GCAGAGGAGGTG	CCACTGCAAAAG
chr8	125906764		5	GAGGAGGAGGAG	CTACAGCAAGAG
chr1	24397474		4	GATGAGGACGGG	CAACAGCAAAAG
chr2	108698300		5	GGTGAGGAGGGG	TAATTTCAAAAG
chr11	46466237		6	GAGGAGGAAGAC	TCACTGCAAAAG
chr1	1247311		6	GACGAGGAGCTG	CAGAGGCAAAAG
chr2	106258357		6	GAAGAGGAGTAC	GAGCTGCAAAGC
chr4	140537415		6	GATGATGAGCAC	CAACAACAACAG

				46698 site	46705 site
chr3	46414562	CCR5*	6	ATCAGGATGAGG	CAAAAGGCTGAAGAGCAT
chr4	55104705		5	TTCAAGATGAAG	CCTAGGTAAGAAGAGGAT
chr6	50841937		7	GTCAAGACAACT	AAAAAGGATGATGAGCAG
chr3	45874336		12	ATCAGGAGCTAC	AGGTAAAGTGAAGAGCAT
chr4	128156339		ND	ND	ND

				46700 site	46705 site
chr3	46414562	CCR5*	6	ATCAGGATGAGGATGACC	CAAAAGGCTGAAGAGCAT
chr4	55104705		5	TTCAAGATGAAGTCACTC	CCTAGGTAAGAAGAGGAT
chr3	45874336		12	ATCAGGAGCTACTTCAGG	AGGTAAAGTGAAGAGCAT
chr4	128156339		ND	ND	ND

Supplementary Table 1: Putative binding sites and putative half-site spacing for each locus listed as an active off-target site in Table 1. Each locus is identified by the coordinate of the median IDLV insertion event. Colored bases highlight mismatches with the intended target sequence. Red bases are mismatched relative to both the intended sequence and SELEX preference. Blue bases are mismatched relative to the intended target but are biochemically preferred as indicated by SELEX data. Such matches to the preferred base can explain the differential activity at many sites between the 46693:46697 ZNF pair and the 46693:46696 pair. The locations of the

indels were used to identify the ZFN cleavage site and thus to identify the putative ZFN binding sites- sites labeled as “ND” did not have a pattern of indels that indicated true ZFN cleavage and thus did not allow the determination of putative ZFN binding sites.

Supplementary Notes

Supplementary Note 1. Overview of the B1H system

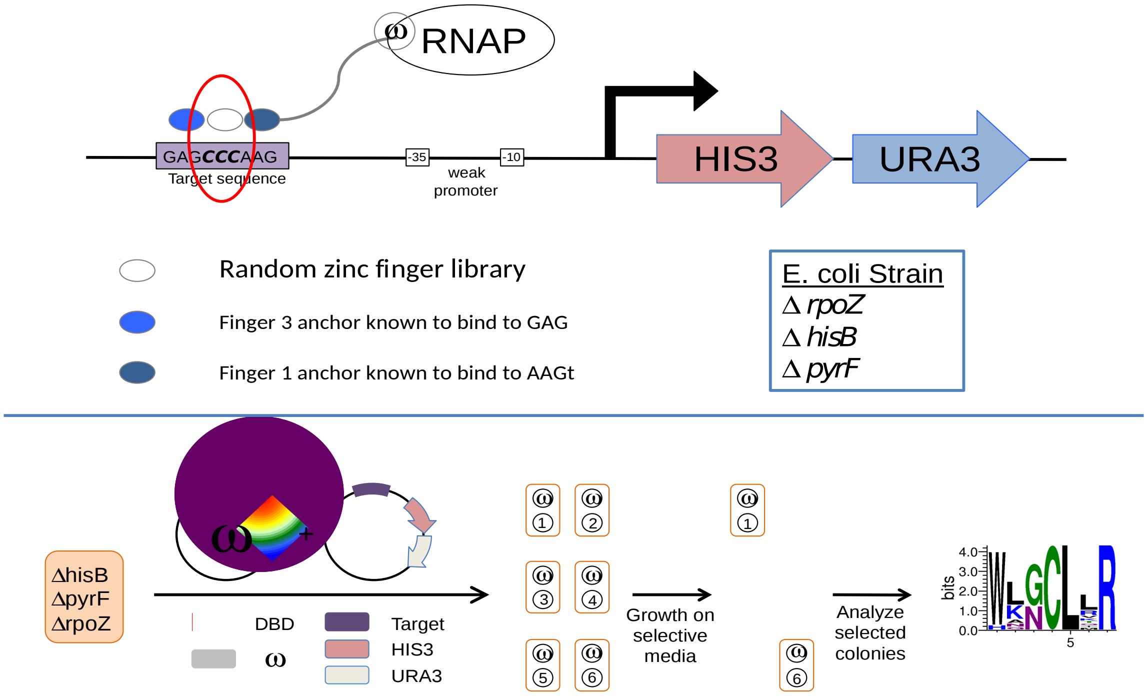

Fundamentally the B1H system allows the investigation of a molecular interaction by making survival of the bacteria dependent on activation of the reporter, in this case HIS3 (selection of URA3 activation is also possible but in this context, its presence is purely historical). This is achieved by first knocking out the bacterial equivalent of HIS3 (*hisB*) and driving HIS3 expression with a weak promoter that only leads to expression above background when RNA Polymerase is actively recruited. In this way, when cells are grown on media that lacks histidine, survival is dependent on activation of this reporter. We are also able to control the required level of HIS3 activation with a competitive inhibitor of HIS3, 3-aminotriazole (3-AT). The more 3AT in the media, the more HIS3 expression required for survival. Ultimately, the strength of the interaction we are assaying will determine how much RNAP is recruited to the promoter, how much HIS3 is then expressed, and finally whether the cells survive the growth conditions.

For recruitment of RNAP, a DBD is expressed as a direct fusion to the omega subunit of RNAP. We have knocked out the endogenous omega (*rpoZ*) to improve the sensitivity of this system. In the example, we are selecting zinc fingers from a library (rainbow oval) to find amino acid combinations that bind to “CCC” as indicated by the red circle. To position the library directly over the 3-nt target of interest, we surround the zinc finger library with two fingers of known specificity (GAG and AAG binding fingers 3 and 1, respectively). These fingers function as anchors to position the library properly. These 3-fingered proteins, with a variable center finger, are

expressed as a fusion to omega. In this way, only library members that interact with “CCC” will recruit the polymerase and activate HIS3.

In practice, we transform two plasmids into our selection strain; one that expresses the zinc finger library - omega fusion, and the reporter plasmid that has been modified with the binding site we are interested in. By limiting the amount of library vector in the transformation we attempt to ensure that a single member of the library gets into each cell. In this way the cell acts as a compartment, compartmentalizing the target sequence of interest with a single member of the protein library. Therefore each cell is its own experiment testing a single pairing of protein and DNA. If this pairing offers a complementary interaction, the reporter is activated and the cells will survive the selective conditions. Finally, the coding sequence from the library region of the expression vector is sequenced from the surviving colonies. In this way we are able to uncover what amino acid profiles have been enriched to bind our target sequence of interest.

Supplementary Note 2. Annotated pGHUC Sequence

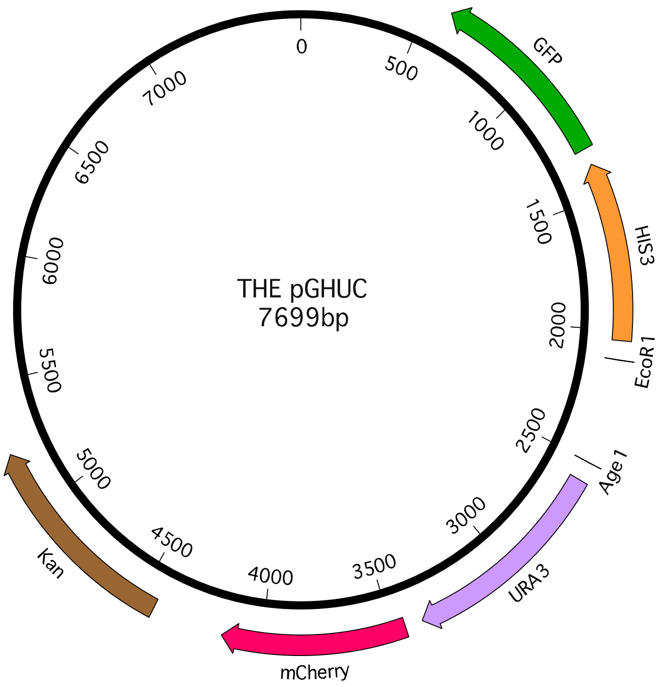

Position 1 promoter (HIS3/GFP)

…GCGGCCGC – target – *GAATTC*-TTTACACTTTATGCTTCCGGCTCGTATGTTG
Not1 EcoR1 -35box -10box

TGTCGACCGAGCGGATAACAATTTCACACAGGAAACAGCT – ATG □ HIS3

Position 2 promoter (URA3/mCherry)

…*GGCGCGCC* – target – ACCGGT-TTTACATCCTATGGCCTTTCGGCTTATGTTC
Asc1 Age1 -35box -10box

CTCAAGTAATACGCTACTGGTAGCCACACAGGAAACAGCT – ATG □ URA3

pGHUC Sequence

GACGCGCCCTGTAGCGGCGCATTAAGCGCGGCGGGTGTGGTGGTTACGCGCAGCGTGACC
GCTACACTTGCCAGCGCCCTAGCGCCCGCTCCTTTCGCTTTCTTCCCTTCCTTTCTCGCC
ACGTTCGCCGGCTTTCCCCGTCAAGCTCTAAATCGGGGGCTCCCTTTAGGGTTCCGATTT
AGTGCTTTACGGCACCTCGACCCCAAAAAACTTGATTAGGGTGATGGTTCACGTAGTGGG

CCATCGCCCTGATAGACGGTTTTTCGCCCTTTGACGTTGGAGTCCACGTTCTTTAATAGT
GGACTCTTGTTCCAAACTGGAACAACACTCAACCCTATCTCGGTCTATTCTTTTGATTTA
TAAGGGATTTTGCCGATTTCGGCCTATTGGTTAAAAAATGAGCTGATTTAACAAAAATTT
AACGCGAATTTTAACAAAATATTAACGCTTACAATTTAGCTCGAGTATAAACGCAGAAAG
GCCCACCCGAAGGTGAGCCAGTGTGACTCTAGTAGAGAGCGTTCACCGACAAACAACAGA
TAAAACGAAAGGCCCAGTCTTTCGACTGAGCCTTTCGTTTTATTTGATGCCTGGCTATTA
TTTGTACAATTCATCCATACCATGGGTAATACCAGCAGCAGTAACAAATTCTAACAAGAC
CATGTGGTCTCTCTTTTCGTTTGGATCTTTGGATAAGGCAGATTGAGTGGATAAGTAATG
GTTGTCTGGTAACAAGACTGGACCATCACCAATTGGAGTATTTTGTTGATAATGGTCAGC
TAATTGAACAGAACCATCTTCAATGTTGTGTCTAATTTTGAAGTTAACTTTGATACCATT
CTTTTGTTTGTCAGCCATGATGTAAACATTGTGAGAGTTATAGTTGTATTCCAATTTGTG
ACCTAAAATGTTACCATCTTCTTTAAAATCAATACCTTTTAATTCGATTCTATTAACTAA
GGTATCACCTTCAAACTTGACTTCAGCTCTGGTCTTGTAGTTACCGTCATCTTTGAAAAA
AATAGTTCTTTCTTGAACATAACCTTCTGGCATGGCAGACTTGAAAAAGTCATGTTGTTT
CATATGATCTGGGTATCTTGAAAAACATTGAACACCATAAGTTAAAGTAGTGACTAAGGT
TGGCCATGGAACTGGCAATTTACCAGTAGTACAAATAAATTTTAAGGTCAATTTACCGTA
AGTAGCATCACCTTCACCTTCACCGGAGACAGAAAATTTGTGACCATTAACATCACCATC
TAATTCAACCAAAATTGGGACAACACCAGTGAATAATTCTTCACCTTTAGACATAGCTGA
CCTCCTTATTAAAGTTAAACAAAAGGATCCCTGCAGCTTTAAATAATCGGTGTCACTACA
TAAGAACACCTTTGGTGGAGGGAACATCGTTGGTACCATTGGGCGAGGTGGCTTCTCTTA
TGGCAACCGCAAGAGCCTTGAACGCACTCTCACTACGGTGATGATCATTCTTGCCTCGCA
GACAATCAACGTGGAGGGTAATTCTGCTAGCCTCTGCAAAGCTTTCAAGAAAATGCGGGA
TCATCTCGCAAGAGAGATCTCCTACTTTCTCCCTTTGCAAACCAAGTTCGACAACTGCGT
ACGGCCTGTTCGAAAGATCTACCACCGCTCTGGAAAGTGCCTCATCCAAAGGCGCAAATC
CTGATCCAAACCTTTTTACTCCACGCACGGCCCCTAGGGCCTCTTTAAAAGCTTGACCGA
GAGCAATCCCGCAGTCTTCAGTGGTGTGATGGTCGTCTATGTGTAAGTCACCAATGCACT
CAACGATTAGCGACCAGCCGGAATGCTTGGCCAGAGCATGTATCATATGGTCCAGAAACC
CTATACCTGTGTGGACGTTAATCACTTGCGATTGTGTGGCCTGTTCTGCTACTGCTTCTG
CCTCTTTTTCTGGGAAGATCGAGTGCTCTATCGCTAGGGGACCACCCTTTAAAGAGATCG
CAATCTGAATCTTGGTTTCATTTGTAATACGCTTTACTAGGGCTTTCTGCTCTGTCATAG
CTGTTTCCTGTGTGAAATTGTTATCCGCTCGGTCGACACAACATACGAGCCGGAAGCATA
AAGTGTAAAGAATTCGTTACGCCCACGCGGCCGCCGGTTTTGTACACGCGTTTGAAGA
CGAAAGGGCCTCGTGATACGCCTATTTTTATAGGTTAATGTCATGAGCGGATACATATTT
GAATGTATTTAGAAAAATAAACAAAAGAGTTTGTAGAAACGCAAAAAGGCCATCCGTCAG
GATGGCCTTCTGCTTAATTTGATGCCTGGCAGTTTATGGCGGGCGTCCTGCCCGCCACCC
TCCGGGCCGTTGCTTCGCAACGTTCAAATCCGCTCCCGGCGGATTTGTCCTACTCAGGAG
AGCGTTCACCGACAAACAACAGATAAAACGAAAGGCCCAGTCTTTCGACTGAGCCTTTCG
TTTTATTTGATGCCTGGCGCGCCAAGCGTGGGCGTCGACCGGTTTTACATCCTATGGCCT
TTCGGCTTATGTTCCTCAAGTAATACGCTACTGGTAGCCACACAGGAAACAGCTATGTCG
AAAGCTACATATAAGGAACGTGCTGCTACTCATCCTAGTCCTGTTGCTGCCAAGCTATTT
AATATCATGCACGAAAAGCAAACAAACTTGTGTGCTTCATTGGATGTTCGTACCACCAAG
GAATTACTGGAGTTAGTTGAAGCATTAGGTCCCAAAATTTGTTTACTAAAAACACATGTG
GATATCTTGACTGATTTTTCCATGGAGGGCACAGTTAAGCCGCTAAAGGCATTATCCGCC
AAGTACAATTTTTTACTCTTCGAAGACAGAAAATTTGCTGACATTGGTAATACAGTCAAA
TTGCAGTACTCTGCGGGTGTATACAGAATAGCAGAATGGGCAGACATTACGAATGCACAC
GGTGTGGTGGGCCCAGGTATTGTTAGCGGTTTGAAGCAGGCGGCAGAAGAAGTAACAAAG

GAACCTAGAGGCCTTTTGATGTTAGCAGAATTGTCATGCAAGGGCTCCCTATCTACTGGA
GAATATACTAAGGGTACTGTTGACATTGCGAAGAGCGACAAAGATTTTGTTATCGGCTTT
ATTGCTCAAAGAGACATGGGTGGAAGAGATGAAGGTTACGATTGGTTGATTATGACACCC
GGTGTGGGTTTAGATGACAAGGGAGACGCATTGGGTCAACAGTATAGAACCGTGGATGAT
GTGGTCTCTACAGGATCTGACATTATTATTGTTGGAAGAGGACTATTTGCAAAGGGAAGG
GATGCTAAGGTAGAGGGTGAACGTTACAGAAAAGCAGGCTGGGAAGCATATTTGAGAAGA
TGCGGCCAGCAAAACTAATAGTGACACCGATTATTTAAAGCTGCAGCACCGGCGTTTTGT
TTAACTTTAATAAGGAGGTCAGCTATGGTGAGCAAGGGCGAGGAGGATAACATGGCCATC
ATCAAGGAGTTCATGCGCTTCAAGGTGCACATGGAGGGCTCCGTGAACGGCCACGAGTTC
GAGATCGAGGGCGAGGGCGAGGGCCGCCCCTACGAGGGCACCCAGACCGCCAAGCTGAAG
GTGACCAAGGGTGGCCCCCTGCCCTTCGCCTGGGACATCCTGTCCCCTCAGTTCATGTAC
GGCTCCAAGGCCTACGTGAAGCACCCCGCCGACATCCCCGACTACTTGAAGCTGTCCTTC
CCCGAGGGCTTCAAGTGGGAGCGCGTGATGAACTTCGAGGACGGCGGCGTGGTGACCGTG
ACCCAGGACTCCTCCCTGCAGGACGGCGAGTTCATCTACAAGGTGAAGCTGCGCGGCACC
AACTTCCCCTCCGACGGCCCCGTAATGCAGAAGAAGACCATGGGCTGGGAGGCCTCCTCC
GAGCGGATGTACCCCGAGGACGGCGCCCTGAAGGGCGAGATCAAGCAGAGGCTGAAGCTG
AAGGACGGCGGCCACTACGACGCTGAGGTCAAGACCACCTACAAGGCCAAGAAGCCCGTG
CAGCTGCCCGGCGCCTACAACGTCAACATCAAGTTGGACATCACCTCCCACAACGAGGAC
TACACCATCGTGGAACAGTACGAACGCGCCGAGGGCCGCCACTCCACAGGAGGCATGGAC
GAGCTGTACAAGTAATGATAGCCAGGCATCAAATAAAACGAAAGGCTCAGTCGAAAGACT
GGGCCTTTCGTTTTATCTGTTGTTTGTCGGTGAACGCTCTCTACTAGAGTCACACTGGCT
CACCTTCGGGTGGGCCTTTCTGCGTTTATAGACGTCGGAATTGCCAGCTGGGGCGCCCTC
TGGTAAGGTTGGGAAGCCCTGCAAAGTAAACTGGATGGCTTTCTTGCCGCCAAGGATCTG
ATGGCGCAGGGGATCAAGATCTGATCAAGAGACAGGATGAGGATCGTTTCGCATGATTGA
ACAAGATGGATTGCACGCAGGTTCTCCGGCCGCTTGGGTGGAGAGGCTATTCGGCTATGA
CTGGGCACAACAGACAATCGGCTGCTCTGATGCCGCCGTGTTCCGGCTGTCAGCGCAGGG
GCGCCCGGTTCTTTTTGTCAAGACCGACCTGTCCGGTGCCCTGAATGAACTGCAGGACGA
GGCAGCGCGGCTATCGTGGCTGGCCACGACGGGCGTTCCTTGCGCAGCTGTGCTCGACGT
TGTCACTGAAGCGGGAAGGGACTGGCTGCTATTGGGCGAAGTGCCGGGGCAGGATCTCCT
GTCATCTCACCTTGCTCCTGCCGAGAAAGTATCCATCATGGCTGATGCAATGCGGCGGCT
GCATACGCTTGATCCGGCTACCTGCCCATTCGACCACCAAGCGAAACATCGCATCGAGCG
AGCACGTACTCGGATGGAAGCCGGTCTTGTCGATCAGGATGATCTGGACGAAGAGCATCA
GGGGCTCGCGCCAGCCGAACTGTTCGCCAGGCTCAAGGCGCGCATGCCCGACGGCGAGGA
TCTCGTCGTGACCCATGGCGATGCCTGCTTGCCGAATATCATGGTGGAAAATGGCCGCTT
TTCTGGATTCATCGACTGTGGCCGGCTGGGTGTGGCGGACCGCTATCAGGACATAGCGTT
GGCTACCCGTGATATTGCTGAAGAGCTTGGCGGCGAATGGGCTGACCGCTTCCTCGTGCT
TTACGGTATCGCCGCTCCCGATTCGCAGCGCATCGCCTTCTATCGCCTTCTTGACGAGTT
CTTCTGAGCGGGACTCTGGGGTTCGAGAGCTCGCTTGGACTCCTGTTGATAGATCCAGTA
ATGACCTCAGAACTCCATCTGGATTTGTTCAGAACGCTCGGTTGCCGCCGGGCGTTTTTT
ATTGGTGAGAATCCAAGCACTAGGGACAGTAAGACGGGTAAGCCTGTTGATGATACCGCT
GCCTTACTGGGTGCATTAGCCAGTCTGAATGACCTGTCACGGGATAATCCGAAGTGGTCA
GACTGGAAAATCAGAGGGCAGGAACTGCTGAACAGCAAAAAGTCAGATAGCACCACATAG
CAGACCCGCCATAAAACGCCCTGAGAAGCCCGTGACGGGCTTTTCTTGTATTATGGGTAG
TTTCCTTGCATGAATCCATAAAAGGCGCCTGTAGTGCCATTTACCCCCATTCACTGCCAG
AGCCGTGAGCGCAGCGAACTGAATGTCACGAAAAAGACAGCGACTCAGGTGCCTGATGGT
CGGAGACAAAAGGAATATTCAGCGATTTGCCCGAGCTTGCGAGGGTGCTACTTAAGCCTT

TAGGGTTTTAAGGTCTGTTTTGTAGAGGAGCAAACAGCGTTTGCGACATCCTTTTGTAAT
ACTGCGGAACTGACTAAAGTAGTGAGTTATACACAGGGCTGGGATCTATTCTTTTTATCT
TTTTTTATTCTTTCTTTATTCTATAAATTATAACCACTTGAATATAAACAAAAAAAACAC
ACAAAGGTCTAGCGGAATTTACAGAGGGTCTAGCAGAATTTACAAGTTTTCCAGCAAAGG
TCTAGCAGAATTTACAGATACCCACAACTCAAAGGAAAAGGACTAGTAATTATCATTGAC
TAGCCCATCTCAATTGGTATAGTGATTAAAATCACCTAGACCAATTGAGATGTATGTCTG
AATTAGTTGTTTTCAAAGCAAATGAACTAGCGATTAGTCGCTATGACTTAACGGAGCATG
AAACCAAGCTAATTTTATGCTGTGTGGCACTACTCAACCCCACGATTGAAAACCCTACAA
GGAAAGAACGGACGGTATCGTTCACTTATAACCAATACGCTCAGATGATGAACATCAGTA
GGGAAAATGCTTATGGTGTATTAGCTAAAGCAACCAGAGAGCTGATGACGAGAACTGTGG
AAATCAGGAATCCTTTGGTTAAAGGCTTTGAGATTTTCCAGTGGACAAACTATGCCAAGT
TCTCAAGCGAAAAATTAGAATTAGTTTTTAGTGAAGAGATATTGCCTTATCTTTTCCAGT
TAAAAAAATTCATAAAATATAATCTGGAACATGTTAAGTCTTTTGAAAACAAATACTCTA
TGAGGATTTATGAGTGGTTATTAAAAGAACTAACACAAAAGAAAACTCACAAGGCAAATA
TAGAGATTAGCCTTGATGAATTTAAGTTCATGTTAATGCTTGAAAATAACTACCATGAGT
TTAAAAGGCTTAACCAATGGGTTTTGAAACCAATAAGTAAAGATTTAAACACTTACAGCA
ATATGAAATTGGTGGTTGATAAGCGAGGCCGCCCGACTGATACGTTGATTTTCCAAGTTG
AACTAGATAGACAAATGGATCTCGTAACCGAACTTGAGAACAACCAGATAAAAATGAATG
GTGACAAAATACCAACAACCATTACATCAGATTCCTACCTACATAACGGACTAAGAAAAA
CACTACACGATGCTTTAACTGCAAAAATTCAGCTCACCAGTTTTGAGGCAAAATTTTTGA
GTGACATGCAAAGTAAGTATGATCTCAATGGTTCGTTCTCATGGCTCACGCAAAAACAAC
GAACCACACTAGAGAACATACTGGCTAAATACGGAAGGATCTGAGGTTCTTATGGCTCTT
GTATCTATCAGTGAAGCATCAAGACTAACAAACAAAAGTAGAACAACTGTTCACCGTTAC
ATATCAAAGGGAAAACTGTCCATATGCACAGATGAAAACGGTGTAAAAAAGATAGATACA
TCAGAGCTTTTACGAGTTTTTGGTGCATTCAAAGCTGTTCACCATGAACAGATCGACAAT
GTAACAGATGAACAGCATGTAACACCTAATAGAACAGGTGAAACCAGTAAAACAAAGCAA
CTAGAACATGAAATTGAACACCTGAGACAACTTGTTACAGCTCAACAGTCACACATAGAC
AGCCTGAAACAGGCGATGCTGCTTATCGAATCAAAGCTGCCGACAACACGGGAGCCAGTG
ACGCCTCCCGTGGGGAAAAAATCATGGCAATTCTGGAAGAAATAGCGCTTTCAGCCGGCA
AACCGGCTGAAGCCGGATCTGCGATTCTGATAACAAACTAGCAACACCAGAACAGCCCGT
TTGCGGGCAGCAAAACCCGTACCCTAGGTCTAGGGCGGCGGATTTGTCCTACTCAGGAGA
GCGTTCACCGACAAACAACAGATAAAACGAAAGGCCCAGTCTTTCGACTGAGCCTTTCGT
TTTATTTGATGCCTCTAGA

Supplementary Note 3. Omega-zinc finger expression vector (Zif268 example)

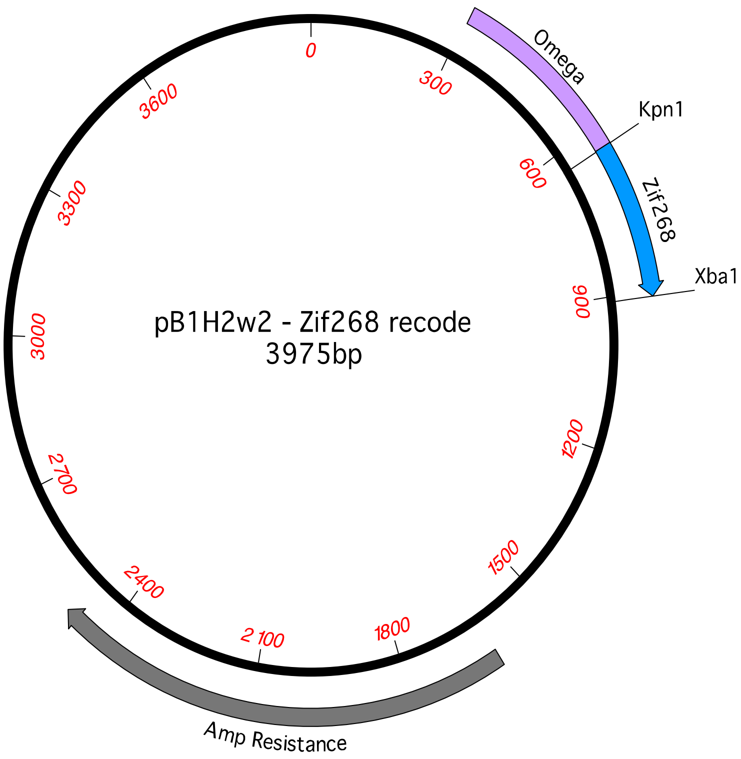

pB1H2w2-Zif268 recode sequence

(Omega-zinc finger fusion driving by mutant UV5 promoter, also referred to as “UV2”)

GAATTCCGGGCTTTACACTTTATGCTTCCGGCTCGCATTATGTGTCGACTTGTGAGCGGA
TAACAATTTCACACAGGAAACAGCTATGCCTCAACAGCAGCAAATGCAACCTCCCAATTC
AAGTGCGGACAACAACCCTTTGCAACAGCAATCATCACAAAATACCGTACCAAACGTCCT
CAACCAAATTAACCAAATCTTTTCTCCAGAGGAGCAACGCAGCTTATTACAAGAAGCCAT
CGAAACCTGCAAGAATTTTGAAAAAACACAATTGTAAGTCTCGGCTCCAAGAAGGAGATA
TACCCATGGCACGCGTAACTGTTCAGGACGCTGTAGAGAAAATTGGTAACCGTTTTGACC
TGGTACTGGTCGCCGCGCGTCGCGCTCGTCAGATGCAGGTAGGCGGAAAGGACCCGCTCG
TACCGGAAGAAAACGATAAAACCACTGTAATCGCGCTGCGCGAAATCGAAGAAGGTCTGA
TCAACAACCAGATCCTCGACGTTCGCGAACGCCAGGAACAGCAAGAGCAGGAAGCCGCTG
AATTACAAGCCGTTACCGCTATTGCTGAAGGTCGTGCGGCCGCGGACTACAAGGATGACG
ACGACAAGTTCCGGACCGGTTCCAAGACACCCCCCCATGGTACCGAGCGCCCATATGCTT
GCCCTGTCGAGTCCTGCGATCGCCGCTTTTCTCGCTCGGATGAGCTTACCCGCCATATCC
GCATCCATACCGGTCAGAAGCCCTTCCAGTGTCGAATCTGCATGCGTAACTTCAGTCGTA
GTGACCACCTTACCACCCACATCCGCACCCACACCGGCGAGAAGCCTTTTGCCTGTGACA
TTTGTGGGAGGAAGTTTGCCAGGAGTGATGAACGCAAGAGGCATACCAAAATCCATCTCC

GCGGATCCTAAGCCTAGGTCTAGAGACTAGAAAAAGGCCGACAAGTCCCGCTCCGCTGAA
GATCCTGGCGTAATAGCGAAGAGGCCCGCACCGATCGCCCTTCCCAACAGTTGCGCAGCC
TGAATGGCGAATGGGACGCGCCCTGTAGCGGCGCATTAAGCGCGGCGGGTGTGGTGGTTA
CGCGCAGCGTGACCGCTACACTTGCCAGCGCCCTAGCGCCCGCTCCTTTCGCTTTCTTCC
CTTCCTTTCTCGCCACGTTCGCCGGCTTTCCCCGTCAAGCTCTAAATCGGGGGCTCCCTT
TAGGGTTCCGATTTAGTGCTTTACGGCACCTCGACCCCAAAAAACTTGATTAGGGTGATG
GTTCACGTAGTGGGCCATCGCCCTGATAGACGGTTTTTCGCCCTTTGACGTTGGAGTCCA
CGTTCTTTAATAGTGGACTCTTGTTCCAAACTGGAACAACACTCAACCCTATCTCGGTCT
ATTCTTTTGATTTATAAGGGATTTTGCCGATTTCGGCCTATTGGTTAAAAAATGAGCTGA
TTTAACAAAAATTTAACGCGAATTTTAACAAAATATTAACGCTTACAATTTAGGTGGCAC
TTTTCGGGGAAATGTGCGCGGAACCCCTATTTGTTTATTTTTCTAAATACATTCAAATAT
GTATCCGCTCATGAGACAATAACCCTGATAAATGCTTCAATAATATTGAAAAAGGAAGAG
TATGAGTATTCAACATTTCCGTGTCGCCCTTATTCCCTTTTTTGCGGCATTTTGCCTTCC
TGTTTTTGCTCACCCAGAAACGCTGGTGAAAGTAAAAGATGCTGAAGATCAGTTGGGTGC
ACGAGTGGGTTACATCGAACTGGATCTCAACAGCGGTAAGATCCTTGAGAGTTTTCGCCC
CGAAGAACGTTTTCCAATGATGAGCACTTTTAAAGTTCTGCTATGTGGCGCGGTATTATC
CCGTATTGACGCCGGGCAAGAGCAACTCGGTCGCCGCATACACTATTCTCAGAATGACTT
GGTTGAGTACTCACCAGTCACAGAAAAGCATCTTACGGATGGCATGACAGTAAGAGAATT
ATGCAGTGCTGCCATAACCATGAGTGATAACACTGCGGCCAACTTACTTCTGACAACGAT
CGGAGGACCGAAGGAGCTAACCGCTTTTTTGCACAACATGGGGGATCATGTAACTCGCCT
TGATCGTTGGGAACCGGAGCTGAATGAAGCCATACCAAACGACGAGCGTGACACCACGAT
GCCTGTAGCAATGGCAACAACGTTGCGCAAACTATTAACTGGCGAACTACTTACTCTAGC
TTCCCGGCAACAATTAATAGACTGGATGGAGGCGGATAAAGTTGCAGGACCACTTCTGCG
CTCGGCCCTTCCGGCTGGCTGGTTTATTGCTGATAAATCTGGAGCCGGTGAGCGTGGGTC
TCGCGGTATCATTGCAGCACTGGGGCCAGATGGTAAGCCCTCCCGTATCGTAGTTATCTA
CACGACGGGGAGTCAGGCAACTATGGATGAACGAAATAGACAGATCGCTGAGATAGGTGC
CTCACTGATTAAGCATTGGTAACTGTCAGACCAAGTTTACTCATATATACTTTAGATTGA
TTTAAAACTTCATTTTTAATTTAAAAGGATCTAGGTGAAGATCCTTTTTGATAATCTCAT
GACCAAAATCCCTTAACGTGAGTTTTCGTTCCACTGAGCGTCAGACCCCGTAGAAAAGAT
CAAAGGATCTTCTTGAGATCCTTTTTTTCTGCGCGTAATCTGCTGCTTGCAAACAAAAAA
ACCACCGCTACCAGCGGTGGTTTGTTTGCCGGATCAAGAGCTACCAACTCTTTTTCCGAA
GGTAACTGGCTTCAGCAGAGCGCAGATACCAAATACTGTCCTTCTAGTGTAGCCGTAGTT
AGGCCACCACTTCAAGAACTCTGTAGCACCGCCTACATACCTCGCTCTGCTAATCCTGTT
ACCAGTGGCTGCTGCCAGTGGCGATAAGTCGTGTCTTACCGGGTTGGACTCAAGACGATA
GTTACCGGATAAGGCGCAGCGGTCGGGCTGAACGGGGGGTTCGTGCACACAGCCCAGCTT
GGAGCGAACGACCTACACCGAACTGAGATACCTACAGCGTGAGCTATGAGAAAGCGCCAC
GCTTCCCGAAGGGAGAAAGGCGGACAGGTATCCGGTAAGCGGCAGGGTCGGAACAGGAGA
GCGCACGAGGGAGCTTCCAGGGGGAAACGCCTGGTATCTTTATAGTCCTGTCGGGTTTCG
CCACCTCTGACTTGAGCGTCGATTTTTGTGATGCTCGTCAGGGGGGCGGAGCCTATGGAA
AAACGCCAGCAACGCGGCCTTTTTACGGTTCCTGGCCTTTTGCTGGCCTTTTGCTCACAT
GTTCTTTCCTGCGTTATCCCCTGATTCTGTGGATAACCGTATTACCGCCTTTGAGTGAGC
TGATACCGCTCGCCGCAGCCGAACGACCGAGCGCAGCGAGTCAGTGAGCGAGGAAGCGGA
AGAGCGCCTGATGCGGTATTTTCTCCTTACGCATCTGTGCGGTATTTCACACCGCATATG
GTGCACTCTCAGTACAATCTGCTCTGATGCCGCATAGTTAAGCCAGTATACACTCCGCTA
TCGCTACGTGACTGGGTCATGGCTGCGCCCCGACACCCGCCAACACCCGCTGACGCGCCC
TGACGGGCTTGTCTGCTCCCGGCATCCGCTTACAGACAAGCTGTGACCGTCTCCGGGAGC

TGCATGTGTCAGAGGTTTTCACCGTCATCACCGAAACGCGCGAGGCAGCTGCGGTAAAGC
TCATCAGCGTGGTCGTGAAGCGATTCACAGATGTCTGCCTGTTCATCCGCGTCCAGCTCG
TTGAGTTTCTCCAGAAGCGTTAATGTCTGGCTTCTGATAAAGCGGGCCATGTTAAGGGCG
GTTTTTTCCTGTTTGGTCACTGATGCCTCCGTGTAAGGGGGATTTCTGTTCATGGGGGTA
ATGATACCGATGAAACGAGAGAGGATGCTCACGATACGGGTTACTGATGATGAACATGCC
CGGTTACTGGAACGG

Supplementary Note 4. Zinc finger template sequences

Four fingered zinc finger pools were assembled as the following sequences, with the intention of overlapping the C-terminal 2 fingers of the "N-4" and the N-terminal 2 fingers of the "C-4" when six fingered proteins were required. However, it was ultimately more time and cost effective to simply design and synthesize to order the 6-fingered proteins based on the selection results. The approach also enabled synthesis to reflect human coding bias. Therefore, many of the zinc fingers used in the cellular studies are based on the selection results but synthesized with optimal coding sequences. NNS bases below represent the helices of the zinc fingers and the bases that were randomized in the original zinc finger pools.

N-4
GGTACCGAGCGCCCATTCCAGTGTCGAATCTGCATGCGTAACTTCAGT*NNSNNSNNSNN*
*SCTGNNSNNS*CACATCCGCACCCACACCGGCGAGAAGCCTTTTGCCTGTGACATTTGTGG
GAGGAAGTTTGCC*NNSNNSNNSNNSCTGNNSNNS*CATACCAAAATCCATACAGGTTCCCA
GAAACCGTTTCAATGCAGGATATGCATGCGTAACTTCAGT*NNSNNSNNSNNSCTGNNSN*
*NS*CACATCCGCACCCACACCGGCGAGAAGCCTTTTGCCTGTGACATTTGTGGGAGGAAGT
TTGCC*NNSNNSNNSNNSCTGNNSNNS*CATACCAAAATCCATTTACGTGGATCCTAAGTCT
AGA

C-4
GGTACCGAGCGCCCATTTCAATGCAGGATATGCATGCGTAACTTCAGT*NNSNNSNNSNN*
*SCTGNNSNNS*CACATCCGCACCCACACCGGCGAGAAGCCTTTTGCCTGTGACATTTGTGG
GAGGAAGTTTGCC*NNSNNSNNSNNSCTGNNSNNS*CATACCAAAATCCATACCGGCAGCCA
GAAGCCATTTCAGTGCCGCATTTGCATGCGTAACTTCAGT*NNSNNSNNSNNSCTGNNSN*
*NS*CACATCCGCACCCACACCGGCGAGAAGCCTTTTGCCTGTGACATTTGTGGGAGGAAGT
TTGCC*NNSNNSNNSNNSCTGNNSNNS*CATACCAAAATCCATTTACGTGGATCCTAAGTCT
AGA

Supplementary Note 5. Position 1 and position 2 target sequences used for counter selections

All binding sites used in the multi-reporter vectors are shown below. Position 1 are placed between Not1 and EcoR1 (*Italic*) with spacing bases (lower case) to position the binding site properly relative to the promoters that drive the reporters. Position

2 binding sites are placed between Asc1 and Age1. Differences between the position 1 and position 2 sequences are noted as red letters. These red letters are the differences between the CCR5 and CCR2 genomic sequences.

Original, left CCR5 target
Position 1 – *GCGGCCGC* – aa GATGAGGATGAC cac – *GAATTC*
Position 2 – *GGCGCGCC* – aa GATGAGGA**C**GAC cac - *ACCGGT*

Original, right CCR5 target
Position 1 – *GCGGCCGC* – aa AAACTGCAAAAG gac – *GAATTC*
Position 2 – *GGCGCGCC* – aa AAACTGCAAAA**A** gac - *ACCGGT*

Shifted, “N-4” left CCR5 target
Position 1 – *GCGGCCGC* – aa ATGAGGATGACC aac – *GAATTC*
Position 2 – *GGCGCGCC* – aa ATGAGGA**C**GACC ATGAGGA**C**GACC cac - *ACCGGT*
atgacca
Shifted, “C-4” left CCR5 target
Position 1 – *GCGGCCGC* – aa ATCAGGATGAGG aac – *GAATTC*
Position 2 – *GGCGCGCC* – aa ATCA**A**GATGAGG AT**T**AGGATGAGG cac - *ACCGGT*

Shifted, “N-4” right CCR5 target
Position 1 – *GCGGCCGC* – aa GCTGAAGAGCAT gac – *GAATTC*
Position 2 – *GGCGCGCC* – aa GCTGAAG**T**GCAT GCTGAAGAGC**T**T gac - *ACCGGT*

Shifted, “C-4” right CCR5 target
Position 1 – *GCGGCCGC* – aa CAAAAGGCTGAA gac – *GAATTC*
Position 2 – *GGCGCGCC* – aa CAAAA**A**GCTGAA CAAAA**A**GCTGAA gac - *ACCGGT*

Supplementary Note 6. Zinc finger nuclease constructs used for cellular studies

Protein and coding sequences for each construct used for cellular studies are provided below. Underlines highlight ZFN recognition helices.

>44671-CKKR
ATGGACTACAAAGACCATGACGGTGATTATAAAGATCATGACATCGATTACAAGGATGACGATGACAAGATG
GCCCCCAAGAAGAAGAGGAAGGTCGGCATTCATGGGGTACCCGCCGCTATGGCTGAGCGCCCATTCCAGTGT
CGAATCTGCATGCGTAACTTCAGTACAAAGTGGAACCTTGACACCCACATCCGCACCCACACCGGCGAGAAG
CCTTTTGCCTGTGACATTTGTGGGAGGAAGTTTGCCAGGGCCAGCACACTCTGGCACCATACCAAAATCCAT
ACAGGTTCCCAGAAACCGTTTCAATGCAGGATATGTATGCGTAACTTCAGTCGCAAGAGCACACTTGTGGAG
CACATCCGCACCCACACCGGCGAGAAGCCTTTTGCCTGTGACATTTGTGGGAGGAAGTTTGCCCAGCGGGGC
AACCTCAAGACACATACCAAAATCCATCTGCGCGGATCCCAGCTGGTGAAGAGCGAGCTGGAGGAGAAGAAG
TCCGAGCTGCGGCACAAGCTGAAGTACGTGCCCCACGAGTACATCGAGCTGATCGAGATCGCCAGGAACAGC
ACCCAGGACCGCATCCTGGAGATGAAGGTGATGGAGTTCTTCATGAAGGTGTACGGCTACAGGGGAAAGCAC
CTGGGCGGAAGCAGAAAGCCTGACGGCGCCATCTATACAGTGGGCAGCCCCATCGATTACGGCGTGATCGTG
GACACAAAGGCCTACAGCGGCGGCTACAATCTGCCTATCGGCCAGGCCGACGAGATGCAGAGATACGTGAAG
GAGAACCAGACCCGGAATAAGCACATCAACCCCAACGAGTGGTGGAAGGTGTACCCTAGCAGCGTGACCGAG
TTCAAGTTCCTGTTCGTGAGCGGCCACTTCAAGGGCAACTACAAGGCCCAGCTGACCAGGCTGAACCGCAAA
ACCAACTGCAATGGCGCCGTGCTGAGCGTGGAGGAGCTGCTGATCGGCGGCGAGATGATCAAAGCCGGCACC
CTGACACTGGAGGAGGTGCGGCGCAAGTTCAACAACGGCGAGATCAACTTCTGATAA

MDYKDHDGDYKDHDIDYKDDDDKMAPKKKRKVGIHGVPAAMAERPFQCRICMRNFS<u>TKWNLDT</u>HIRTHTGE
KPFACDICGRKFA<u>RASTLWH</u>HTKIHTGSQKPFQCRICMRNFS<u>RKSTLVE</u>HIRTHTGEKPFACDICGRKFA<u>Q</u>
<u>RGNLK</u>THTKIHLRGSQLVKSELEEKKSELRHKLKYVPHEYIELIEIARNSTQDRILEMKVMEFFMKVYGYR

GKHLGGSRKPDGAIYTVGSPIDYGVIVDTKAYSGGYNLPIGQADEMQRYVKENQTRNKHINPNEWWKVYPS
SVTEFKFLFVSGHFKGNYKAQLTRLNRKTNCNGAVLSVEELLIGGEMIKAGTLTLEEVRRKFNNGEINF

>44672-NELD
ATGGACTACAAAGACCATGACGGTGATTATAAAGATCATGACATCGATTACAAGGATGACGATGACAAGATG
GCCCCCAAGAAGAAGAGGAAGGTCGGCATCCACGGGGTACCCGCCGCTATGGCTGAGCGCCCATTCCAGTGT
CGAATCTGCATGCGTAACTTCAGTGACAGGTCGAACCTTCTCAGGCACATCCGCACCCACACCGGCGAGAAG
CCTTTTGCCTGTGACATTTGTGGGAGGAAGTTTGCCTTCTTGGGCAACCTCCGCCGGCATACCAAAATCCAT
ACAGGTTCCCAGAAACCGTTTCAATGCAGGATATGTATGCGTAACTTCAGTACCCAGTTCAACCTTGAGCGG
CACATCCGCACCCACACCGGCGAGAAGCCTTTTGCCTGTGACATTTGTGGGAGGAAGTTTGCCATGCGGGCG
AACCTCAGGCGGCATACCAAAATCCATCTGCGCGGATCCCAGCTGGTGAAGAGCGAGCTGGAGGAGAAGAAG
TCCGAGCTGCGGCACAAGCTGAAGTACGTGCCCCACGAGTACATCGAGCTGATCGAGATCGCCAGGAACAGC
ACCCAGGACCGCATCCTGGAGATGAAGGTGATGGAGTTCTTCATGAAGGTGTACGGCTACAGGGGAAAGCAC
CTGGGCGGAAGCAGAAAGCCTGACGGCGCCATCTATACAGTGGGCAGCCCCATCGATTACGGCGTGATCGTG
GACACAAAGGCCTACAGCGGCGGCTACAATCTGCCTATCGGCCAGGCCGACGAGATGGAGAGATACGTGGAG
GAGAACCAGACCCGGGATAAGCACCTCAACCCCAACGAGTGGTGGAAGGTGTACCCTAGCAGCGTGACCGAG
TTCAAGTTCCTGTTCGTGAGCGGCCACTTCAAGGGCAACTACAAGGCCCAGCTGACCAGGCTGAACCACATC
ACCAACTGCAATGGCGCCGTGCTGAGCGTGGAGGAGCTGCTGATCGGCGGCGAGATGATCAAAGCCGGCACC
CTGACACTGGAGGAGGTGCGGCGCAAGTTCAACAACGGCGAGATCAACTTCAGATCTTGATAA

MDYKDHDGDYKDHDIDYKDDDDKMAPKKKRKVGIHGVPAAMAERPFQCRICMRNFSDRSNLLRHIRTHTGE
KPFACDICGRKFAFLGNLRRHTKIHTGSQKPFQCRICMRNFSTQFNLERHIRTHTGEKPFACDICGRKFAM
RANLRRHTKIHLRGSQLVKSELEEKKSELRHKLKYVPHEYIELIEIARNSTQDRILEMKVMEFFMKVYGYR
GKHLGGSRKPDGAIYTVGSPIDYGVIVDTKAYSGGYNLPIGQADEMERYVEENQTRDKHLNPNEWWKVYPS
SVTEFKFLFVSGHFKGNYKAQLTRLNHITNCNGAVLSVEELLIGGEMIKAGTLTLEEVRRKFNNGEINFRS

>46693-NELD
ATGGACTACAAAGACCATGACGGTGATTATAAAGATCATGACATCGATTACAAGGATGACGATGACAAGATG
GCCCCCAAGAAGAAGAGGAAGGTCGGCATCCACGGGGTACCCGCCGCTATGGCTGAGAGACCCTTTCAGTGT
CGCATCTGTATGCGGAACTTTTCCGACCAGAGCAACCTGACCAGACATATCCGCACACACACTGGAGAAAAG
CCTTTCGCATGTGACATTTGCGGACGCAAGTTTGCTGCCCCCAGCAACCTGTGGAGACACACTAAAATCCAT
ACAGGGTCCCAGAAACCATTCCAATGTAGGATTTGCATGCGCAATTTCTCTACCCTGTACAACCTGACCAGA
CACATTAGGACTCATACAGGGGAGAAACCATTCGCTTGCGATATCTGTGGGAGGAAATTCGCATTCCTGGGC
AACCTGACCAGACATACAAAGATTCACCTGAGAGGATCCCAGCTGGTGAAGAGCGAGCTGGAGGAGAAGAAG
TCCGAGCTGCGGCACAAGCTGAAGTACGTGCCCCACGAGTACATCGAGCTGATCGAGATCGCCAGGAACAGC
ACCCAGGACCGCATCCTGGAGATGAAGGTGATGGAGTTCTTCATGAAGGTGTACGGCTACAGGGGAAAGCAC
CTGGGCGGAAGCAGAAAGCCTGACGGCGCCATCTATACAGTGGGCAGCCCCATCGATTACGGCGTGATCGTG
GACACAAAGGCCTACAGCGGCGGCTACAATCTGCCTATCGGCCAGGCCGACGAGATGGAGAGATACGTGGAG
GAGAACCAGACCCGGGATAAGCACCTCAACCCCAACGAGTGGTGGAAGGTGTACCCTAGCAGCGTGACCGAG
TTCAAGTTCCTGTTCGTGAGCGGCCACTTCAAGGGCAACTACAAGGCCCAGCTGACCAGGCTGAACCACATC
ACCAACTGCAATGGCGCCGTGCTGAGCGTGGAGGAGCTGCTGATCGGCGGCGAGATGATCAAAGCCGGCACC
CTGACACTGGAGGAGGTGCGGCGCAAGTTCAACAACGGCGAGATCAACTTCAGATCTTGATAA

MDYKDHDGDYKDHDIDYKDDDDKMAPKKKRKVGIHGVPAAMAERPFQCRICMRNFSDQSNLTRHIRTHTGE
KPFACDICGRKFAAPSNLWRHTKIHTGSQKPFQCRICMRNFSTLYNLTRHIRTHTGEKPFACDICGRKFAF
LGNLTRHTKIHLRGSQLVKSELEEKKSELRHKLKYVPHEYIELIEIARNSTQDRILEMKVMEFFMKVYGYR
GKHLGGSRKPDGAIYTVGSPIDYGVIVDTKAYSGGYNLPIGQADEMERYVEENQTRDKHLNPNEWWKVYPS
SVTEFKFLFVSGHFKGNYKAQLTRLNHITNCNGAVLSVEELLIGGEMIKAGTLTLEEVRRKFNNGEINFRS

>46694-NELD
ATGGACTACAAAGACCATGACGGTGATTATAAAGATCATGACATCGATTACAAGGATGACGATGACAAGATG
GCCCCCAAGAAGAAGAGGAAGGTCGGCATCCACGGGGTACCCGCCGCTATGGCTGAGAGACCCTTTCAGTGT
CGCATCTGTATGCGGAACTTTTCCTGGCAGGCCAACCTGCTGAGACATATCCGCACACACACTGGAGAAAAG
CCTTTCGCATGTGACATTTGCGGACGCAAGTTTGCTTTCGCCAGCAACCTGATCAGACACACTAAAATCCAT
ACAGGGTCCCAGAAACCATTCCAATGTAGGATTTGCATGCGCAATTTCTCTACCCTGTGGAGCCTGACCAGA

CACATTAGGACTCATACAGGGGAGAAACCATTCGCTTGCGATATCTGTGGGAGGAAATTCGCAACCAAGCAG
AACCTGCAGAGACATACAAAGATTCACCTGAGAGGATCCCAGCTGGTGAAGAGCGAGCTGGAGGAGAAGAAG
TCCGAGCTGCGGCACAAGCTGAAGTACGTGCCCCACGAGTACATCGAGCTGATCGAGATCGCCAGGAACAGC
ACCCAGGACCGCATCCTGGAGATGAAGGTGATGGAGTTCTTCATGAAGGTGTACGGCTACAGGGGAAAGCAC
CTGGGCGGAAGCAGAAAGCCTGACGGCGCCATCTATACAGTGGGCAGCCCCATCGATTACGGCGTGATCGTG
GACACAAAGGCCTACAGCGGCGGCTACAATCTGCCTATCGGCCAGGCCGACGAGATGGAGAGATACGTGGAG
GAGAACCAGACCCGGGATAAGCACCTCAACCCCAACGAGTGGTGGAAGGTGTACCCTAGCAGCGTGACCGAG
TTCAAGTTCCTGTTCGTGAGCGGCCACTTCAAGGGCAACTACAAGGCCCAGCTGACCAGGCTGAACCACATC
ACCAACTGCAATGGCGCCGTGCTGAGCGTGGAGGAGCTGCTGATCGGCGGCGAGATGATCAAAGCCGGCACC
CTGACACTGGAGGAGGTGCGGCGCAAGTTCAACAACGGCGAGATCAACTTCAGATCTTGATAA

MDYKDHDGDYKDHDIDYKDDDDKMAPKKKRKVGIHGVPAAMAERPFQCRICMRNFSWQANLLRHIRTHTGE
KPFACDICGRKFAFASNLIRHTKIHTGSQKPFQCRICMRNFSTLWSLTRHIRTHTGEKPFACDICGRKFAT
KQNLQRHTKIHLRGSQLVKSELEEKKSELRHKLKYVPHEYIELIEIARNSTQDRILEMKVMEFFMKVYGYR
GKHLGGSRKPDGAIYTVGSPIDYGVIVDTKAYSGGYNLPIGQADEMERYVEENQTRDKHLNPNEWWKVYPS
SVTEFKFLFVSGHFKGNYKAQLTRLNHITNCNGAVLSVEELLIGGEMIKAGTLTLEEVRRKFNNGEINFRS

>46695-CKKR
ATGGACTACAAAGACCATGACGGTGATTATAAAGATCATGACATCGATTACAAGGATGACGATGACAAGATG
GCCCCCAAGAAGAAGAGGAAGGTCGGCATTCATGGGGTACCCGCCGCTATGGCTGAGAGACCCTTTCAGTGT
CGCATCTGTATGCGGAACTTTTCCAGAAAGGACAACCTGACCCAGCATATCCGCACACACACTGGAGAAAAG
CCTTTCGCATGTGACATTTGCGGACGCAAGTTTGCTAGAGCCAGCACCCTGTGGCACCACACTAAAATCCAT
ACAGGGTCCCAGAAACCATTCCAATGTAGGATTTGCATGCGCAATTTCTCTAGAAAGAGCACCCTGAACGAC
CACATTAGGACTCATACAGGGGAGAAACCATTCGCTTGCGATATCTGTGGGAGGAAATTCGCACAGAAGGGC
AACCTGAACCAGCATACAAAGATTCACCTGAGAGGATCCCAGCTGGTGAAGAGCGAGCTGGAGGAGAAGAAG
TCCGAGCTGCGGCACAAGCTGAAGTACGTGCCCCACGAGTACATCGAGCTGATCGAGATCGCCAGGAACAGC
ACCCAGGACCGCATCCTGGAGATGAAGGTGATGGAGTTCTTCATGAAGGTGTACGGCTACAGGGGAAAGCAC
CTGGGCGGAAGCAGAAAGCCTGACGGCGCCATCTATACAGTGGGCAGCCCCATCGATTACGGCGTGATCGTG
GACACAAAGGCCTACAGCGGCGGCTACAATCTGCCTATCGGCCAGGCCGACGAGATGCAGAGATACGTGAAG
GAGAACCAGACCCGGAATAAGCACATCAACCCCAACGAGTGGTGGAAGGTGTACCCTAGCAGCGTGACCGAG
TTCAAGTTCCTGTTCGTGAGCGGCCACTTCAAGGGCAACTACAAGGCCCAGCTGACCAGGCTGAACCGCAAA
ACCAACTGCAATGGCGCCGTGCTGAGCGTGGAGGAGCTGCTGATCGGCGGCGAGATGATCAAAGCCGGCACC
CTGACACTGGAGGAGGTGCGGCGCAAGTTCAACAACGGCGAGATCAACTTCTGATAA

MDYKDHDGDYKDHDIDYKDDDDKMAPKKKRKVGIHGVPAAMAERPFQCRICMRNFSRKDNLTQHIRTHTGE
KPFACDICGRKFARASTLWHHTKIHTGSQKPFQCRICMRNFSRKSTLNDHIRTHTGEKPFACDICGRKFAQ
KGNLNQHTKIHLRGSQLVKSELEEKKSELRHKLKYVPHEYIELIEIARNSTQDRILEMKVMEFFMKVYGYR
GKHLGGSRKPDGAIYTVGSPIDYGVIVDTKAYSGGYNLPIGQADEMQRYVKENQTRNKHINPNEWWKVYPS
SVTEFKFLFVSGHFKGNYKAQLTRLNRKTNCNGAVLSVEELLIGGEMIKAGTLTLEEVRRKFNNGEINF

>46696-CKKR
ATGGACTACAAAGACCATGACGGTGATTATAAAGATCATGACATCGATTACAAGGATGACGATGACAAGATG
GCCCCCAAGAAGAAGAGGAAGGTCGGCATTCATGGGGTACCCGCCGCTATGGCTGAGAGACCCTTTCAGTGT
CGCATCTGTATGCGGAACTTTTCCACCAAGTGGAACCTGACCACCCATATCCGCACACACACTGGAGAAAAG
CCTTTCGCATGTGACATTTGCGGACGCAAGTTTGCTCAGAAGATCAACCTGACCGCCCACACTAAAATCCAT
ACAGGGTCCCAGAAACCATTCCAATGTAGGATTTGCATGCGCAATTTCTCTAGAAAGTGGGTGCTGGACTGC
CACATTAGGACTCATACAGGGGAGAAACCATTCGCTTGCGATATCTGTGGGAGGAAATTCGCAAACCCCGGC
AGCCTGCACAACCATACAAAGATTCACCTGAGAGGATCCCAGCTGGTGAAGAGCGAGCTGGAGGAGAAGAAG
TCCGAGCTGCGGCACAAGCTGAAGTACGTGCCCCACGAGTACATCGAGCTGATCGAGATCGCCAGGAACAGC
ACCCAGGACCGCATCCTGGAGATGAAGGTGATGGAGTTCTTCATGAAGGTGTACGGCTACAGGGGAAAGCAC
CTGGGCGGAAGCAGAAAGCCTGACGGCGCCATCTATACAGTGGGCAGCCCCATCGATTACGGCGTGATCGTG
GACACAAAGGCCTACAGCGGCGGCTACAATCTGCCTATCGGCCAGGCCGACGAGATGCAGAGATACGTGAAG
GAGAACCAGACCCGGAATAAGCACATCAACCCCAACGAGTGGTGGAAGGTGTACCCTAGCAGCGTGACCGAG
TTCAAGTTCCTGTTCGTGAGCGGCCACTTCAAGGGCAACTACAAGGCCCAGCTGACCAGGCTGAACCGCAAA

ACCAACTGCAATGGCGCCGTGCTGAGCGTGGAGGAGCTGCTGATCGGCGGCGAGATGATCAAAGCCGGCACC
CTGACACTGGAGGAGGTGCGGCGCAAGTTCAACAACGGCGAGATCAACTTCTGATAA

MDYKDHDGDYKDHDIDYKDDDDKMAPKKKRKVGIHGVPAAMAERPFQCRICMRNFSTKWNLTTHIRTHTGE
KPFACDICGRKFAQKINLTAHTKIHTGSQKPFQCRICMRNFSRKWVLDCHIRTHTGEKPFACDICGRKFAN
PGSLHNHTKIHLRGSQLVKSELEEKKSELRHKLKYVPHEYIELIEIARNSTQDRILEMKVMEFFMKVYGYR
GKHLGGSRKPDGAIYTVGSPIDYGVIVDTKAYSGGYNLPIGQADEMQRYVKENQTRNKHINPNEWWKVYPS
SVTEFKFLFVSGHFKGNYKAQLTRLNRKTNCNGAVLSVEELLIGGEMIKAGTLTLEEVRRKFNNGEINF

>46697-CKKR
ATGGACTACAAAGACCATGACGGTGATTATAAAGATCATGACATCGATTACAAGGATGACGATGACAAGATG
GCCCCCAAGAAGAAGAGGAAGGTCGGCATTCATGGGGTACCCGCCGCTATGGCTGAGAGACCCTTTCAGTGT
CGCATCTGTATGCGGAACTTTTCCACCAAGTGGAACCTGACCACCCATATCCGCACACACACTGGAGAAAAG
CCTTTCGCATGTGACATTTGCGGACGCAAGTTTGCTCAGAAGATCAACCTGACCGCCCACACTAAAATCCAT
ACAGGGTCCCAGAAACCATTCCAATGTAGGATTTGCATGCGCAATTTCTCTAGAAAGAGCACCCTGAACGAC
CACATTAGGACTCATACAGGGGAGAAACCATTCGCTTGCGATATCTGTGGGAGGAAATTCGCACAGAAGGGC
AACCTGAACCAGCATACAAAGATTCACCTGAGAGGATCCCAGCTGGTGAAGAGCGAGCTGGAGGAGAAGAAG
TCCGAGCTGCGGCACAAGCTGAAGTACGTGCCCCACGAGTACATCGAGCTGATCGAGATCGCCAGGAACAGC
ACCCAGGACCGCATCCTGGAGATGAAGGTGATGGAGTTCTTCATGAAGGTGTACGGCTACAGGGGAAAGCAC
CTGGGCGGAAGCAGAAAGCCTGACGGCGCCATCTATACAGTGGGCAGCCCCATCGATTACGGCGTGATCGTG
GACACAAAGGCCTACAGCGGCGGCTACAATCTGCCTATCGGCCAGGCCGACGAGATGCAGAGATACGTGAAG
GAGAACCAGACCCGGAATAAGCACATCAACCCCAACGAGTGGTGGAAGGTGTACCCTAGCAGCGTGACCGAG
TTCAAGTTCCTGTTCGTGAGCGGCCACTTCAAGGGCAACTACAAGGCCCAGCTGACCAGGCTGAACCGCAAA
ACCAACTGCAATGGCGCCGTGCTGAGCGTGGAGGAGCTGCTGATCGGCGGCGAGATGATCAAAGCCGGCACC
CTGACACTGGAGGAGGTGCGGCGCAAGTTCAACAACGGCGAGATCAACTTCTGATAA

MDYKDHDGDYKDHDIDYKDDDDKMAPKKKRKVGIHGVPAAMAERPFQCRICMRNFSTKWNLTTHIRTHTGE
KPFACDICGRKFAQKINLTAHTKIHTGSQKPFQCRICMRNFSRKSTLNDHIRTHTGEKPFACDICGRKFAQ
KGNLNQHTKIHLRGSQLVKSELEEKKSELRHKLKYVPHEYIELIEIARNSTQDRILEMKVMEFFMKVYGYR
GKHLGGSRKPDGAIYTVGSPIDYGVIVDTKAYSGGYNLPIGQADEMQRYVKENQTRNKHINPNEWWKVYPS
SVTEFKFLFVSGHFKGNYKAQLTRLNRKTNCNGAVLSVEELLIGGEMIKAGTLTLEEVRRKFNNGEINF

>46698-NELD
ATGGACTACAAAGACCATGACGGTGATTATAAAGATCATGACATCGATTACAAGGATGACGATGACAAGATG
GCCCCCAAGAAGAAGAGGAAGGTCGGCATCCACGGGGTACCCGCCGCTATGGCTGAGAGACCCTTTCAGTGT
CGCATCTGTATGCGGAACTTTTCCAGAAAGGCCCACCTGGTGAACCATATCCGCACACACACTGGAGAAAAG
CCTTTCGCATGTGACATTTGCGGACGCAAGTTTGCTTGGCAGAGCGGCCTGTGCAACCACACTAAAATCCAT
ACAGGGTCCCAGAAACCATTCCAATGTAGGATTTGCATGCGCAATTTCTCTAGAAAGAGCCACCTGGTGGAC
CACATTAGGACTCATACAGGGGAGAAACCATTCGCTTGCGATATCTGTGGGAGGAAATTCGCAAGCGCCAGC
GGCCTGTGCCACCATACAAAGATTCACCTGAGAGGATCCCAGCTGGTGAAGAGCGAGCTGGAGGAGAAGAAG
TCCGAGCTGCGGCACAAGCTGAAGTACGTGCCCCACGAGTACATCGAGCTGATCGAGATCGCCAGGAACAGC
ACCCAGGACCGCATCCTGGAGATGAAGGTGATGGAGTTCTTCATGAAGGTGTACGGCTACAGGGGAAAGCAC
CTGGGCGGAAGCAGAAAGCCTGACGGCGCCATCTATACAGTGGGCAGCCCCATCGATTACGGCGTGATCGTG
GACACAAAGGCCTACAGCGGCGGCTACAATCTGCCTATCGGCCAGGCCGACGAGATGGAGAGATACGTGGAG
GAGAACCAGACCCGGGATAAGCACCTCAACCCCAACGAGTGGTGGAAGGTGTACCCTAGCAGCGTGACCGAG
TTCAAGTTCCTGTTCGTGAGCGGCCACTTCAAGGGCAACTACAAGGCCCAGCTGACCAGGCTGAACCACATC
ACCAACTGCAATGGCGCCGTGCTGAGCGTGGAGGAGCTGCTGATCGGCGGCGAGATGATCAAAGCCGGCACC
CTGACACTGGAGGAGGTGCGGCGCAAGTTCAACAACGGCGAGATCAACTTCAGATCTTGATAA

MDYKDHDGDYKDHDIDYKDDDDKMAPKKKRKVGIHGVPAAMAERPFQCRICMRNFSRKAHLVNHIRTHTGE
KPFACDICGRKFAWQSGLCNHTKIHTGSQKPFQCRICMRNFSRKSHLVDHIRTHTGEKPFACDICGRKFAS
ASGLCHHTKIHLRGSQLVKSELEEKKSELRHKLKYVPHEYIELIEIARNSTQDRILEMKVMEFFMKVYGYR
GKHLGGSRKPDGAIYTVGSPIDYGVIVDTKAYSGGYNLPIGQADEMERYVEENQTRDKHLNPNEWWKVYPS
SVTEFKFLFVSGHFKGNYKAQLTRLNHITNCNGAVLSVEELLIGGEMIKAGTLTLEEVRRKFNNGEINFRS

>46699-NELD
ATGGACTACAAAGACCATGACGGTGATTATAAAGATCATGACATCGATTACAAGGATGACGATGACAAGATG
GCCCCCAAGAAGAAGAGGAAGGTCGGCATCCACGGGGTACCCGCCGCTATGGCTGAGAGACCCTTTCAGTGT
CGCATCTGTATGCGGAACTTTTCCAGAAAGGCCCACCTGGTGGACCATATCCGCACACACACTGGAGAAAAG
CCTTTCGCATGTGACATTTGCGGACGCAAGTTTGCTGCCAGAGCCGGCCTGTGGCAGCACACTAAAATCCAT
ACAGGGTCCCAGAAACCATTCCAATGTAGGATTTGCATGCGCAATTTCTCTAGAAAGGCCAACCTGTACAAC
CACATTAGGACTCATACAGGGGAGAAACCATTCGCTTGCGATATCTGTGGGAGGAAATTCGCAAGCCAGAGC
GGCCTGTGCCACCATACAAAGATTCACCTGAGAGGATCCCAGCTGGTGAAGAGCGAGCTGGAGGAGAAGAAG
TCCGAGCTGCGGCACAAGCTGAAGTACGTGCCCCACGAGTACATCGAGCTGATCGAGATCGCCAGGAACAGC
ACCCAGGACCGCATCCTGGAGATGAAGGTGATGGAGTTCTTCATGAAGGTGTACGGCTACAGGGGAAAGCAC
CTGGGCGGAAGCAGAAAGCCTGACGGCGCCATCTATACAGTGGGCAGCCCCATCGATTACGGCGTGATCGTG
GACACAAAGGCCTACAGCGGCGGCTACAATCTGCCTATCGGCCAGGCCGACGAGATGGAGAGATACGTGGAG
GAGAACCAGACCCGGGATAAGCACCTCAACCCCAACGAGTGGTGGAAGGTGTACCCTAGCAGCGTGACCGAG
TTCAAGTTCCTGTTCGTGAGCGGCCACTTCAAGGGCAACTACAAGGCCCAGCTGACCAGGCTGAACCACATC
ACCAACTGCAATGGCGCCGTGCTGAGCGTGGAGGAGCTGCTGATCGGCGGCGAGATGATCAAAGCCGGCACC
CTGACACTGGAGGAGGTGCGGCGCAAGTTCAACAACGGCGAGATCAACTTCAGATCTTGATAA

MDYKDHDGDYKDHDIDYKDDDDKMAPKKKRKVGIHGVPAAMAERPFQCRICMRNFSRKAHLVDHIRTHTGE
KPFACDICGRKFAARAGLWQHTKIHTGSQKPFQCRICMRNFSRKANLYNHIRTHTGEKPFACDICGRKFAS
QSGLCHHTKIHLRGSQLVKSELEEKKSELRHKLKYVPHEYIELIEIARNSTQDRILEMKVMEFFMKVYGYR
GKHLGGSRKPDGAIYTVGSPIDYGVIVDTKAYSGGYNLPIGQADEMERYVEENQTRDKHLNPNEWWKVYPS
SVTEFKFLFVSGHFKGNYKAQLTRLNHITNCNGAVLSVEELLIGGEMIKAGTLTLEEVRRKFNNGEINFRS

>46700-NELD
ATGGACTACAAAGACCATGACGGTGATTATAAAGATCATGACATCGATTACAAGGATGACGATGACAAGATG
GCCCCCAAGAAGAAGAGGAAGGTCGGCATCCACGGGGTACCCGCCGCTATGGCTGAGAGACCCTTCCAGTGC
AGAATCTGCATGAGAAACTTCAGCGACCCCAGAAGCCTGGTGAACCACATCAGAACCCACACCGGCGAGAAG
CCCTTTGCCTGCGACATCTGCGGCAGAAAGTTCGCCGCCAGAAACGGCCTGTGGCAGCACACCAAGATCCAC
ACCGGCAGCCAGAAGCCCTTTCAGTGTCGCATCTGTATGCGGAACTTTTCCAGAAAGGCCCACCTGGTGAAC
CATATCCGCACACACACTGGAGAAAAGCCTTTCGCATGTGACATTTGCGGACGCAAGTTTGCTTGGCAGAGC
GGCCTGTGCAACCACACTAAAATCCATACAGGGTCCCAGAAACCATTCCAATGTAGGATTTGCATGCGCAAT
TTCTCTAGAAAGAGCCACCTGGTGGACCACATTAGGACTCATACAGGGGAGAAACCATTCGCTTGCGATATC
TGTGGGAGGAAATTCGCAAGCGCCAGCGGCCTGTGCCACCATACAAAGATTCACCTGAGAGGATCCCAGCTG
GTGAAGAGCGAGCTGGAGGAGAAGAAGTCCGAGCTGCGGCACAAGCTGAAGTACGTGCCCCACGAGTACATC
GAGCTGATCGAGATCGCCAGGAACAGCACCCAGGACCGCATCCTGGAGATGAAGGTGATGGAGTTCTTCATG
AAGGTGTACGGCTACAGGGGAAAGCACCTGGGCGGAAGCAGAAAGCCTGACGGCGCCATCTATACAGTGGGC
AGCCCCATCGATTACGGCGTGATCGTGGACACAAAGGCCTACAGCGGCGGCTACAATCTGCCTATCGGCCAG
GCCGACGAGATGGAGAGATACGTGGAGGAGAACCAGACCCGGGATAAGCACCTCAACCCCAACGAGTGGTGG
AAGGTGTACCCTAGCAGCGTGACCGAGTTCAAGTTCCTGTTCGTGAGCGGCCACTTCAAGGGCAACTACAAG
GCCCAGCTGACCAGGCTGAACCACATCACCAACTGCAATGGCGCCGTGCTGAGCGTGGAGGAGCTGCTGATC
GGCGGCGAGATGATCAAAGCCGGCACCCTGACACTGGAGGAGGTGCGGCGCAAGTTCAACAACGGCGAGATC
AACTTCAGATCTTGATAA

MDYKDHDGDYKDHDIDYKDDDDKMAPKKKRKVGIHGVPAAMAERPFQCRICMRNFSDPRSLVNHIRTHTGE
KPFACDICGRKFAARNGLWQHTKIHTGSQKPFQCRICMRNFSRKAHLVNHIRTHTGEKPFACDICGRKFAW
QSGLCNHTKIHTGSQKPFQCRICMRNFSRKSHLVDHIRTHTGEKPFACDICGRKFASASGLCHHTKIHLRG
SQLVKSELEEKKSELRHKLKYVPHEYIELIEIARNSTQDRILEMKVMEFFMKVYGYRGKHLGGSRKPDGAI
YTVGSPIDYGVIVDTKAYSGGYNLPIGQADEMERYVEENQTRDKHLNPNEWWKVYPSSVTEFKFLFVSGHF
KGNYKAQLTRLNHITNCNGAVLSVEELLIGGEMIKAGTLTLEEVRRKFNNGEINFRS

>46701-NELD
ATGGACTACAAAGACCATGACGGTGATTATAAAGATCATGACATCGATTACAAGGATGACGATGACAAGATG
GCCCCCAAGAAGAAGAGGAAGGTCGGCATCCACGGGGTACCCGCCGCTATGGCTGAGAGACCCTTCCAGTGC
AGAATCTGCATGAGAAACTTCAGCGAGAAGAGAGGCCTGCTGAACCACATCAGAACCCACACCGGCGAGAAG

CCCTTTGCCTGCGACATCTGCGGCAGAAAGTTCGCCAGCGGCGCCGGCCTGTGGCAGCACACCAAGATCCAC
ACCGGCAGCCAGAAGCCCTTTCAGTGTCGCATCTGTATGCGGAACTTTTCCAGAAAGGCCCACCTGGTGGAC
CATATCCGCACACACACTGGAGAAAAGCCTTTCGCATGTGACATTTGCGGACGCAAGTTTGCTGCCAGAGCC
GGCCTGTGGCAGCACACTAAAATCCATACAGGGTCCCAGAAACCATTCCAATGTAGGATTTGCATGCGCAAT
TTCTCTAGAAAGGCCAACCTGTACAACCACATTAGGACTCATACAGGGGAGAAACCATTCGCTTGCGATATC
TGTGGGAGGAAATTCGCAAGCCAGAGCGGCCTGTGCCACCATACAAAGATTCACCTGAGAGGATCCCAGCTG
GTGAAGAGCGAGCTGGAGGAGAAGAAGTCCGAGCTGCGGCACAAGCTGAAGTACGTGCCCCACGAGTACATC
GAGCTGATCGAGATCGCCAGGAACAGCACCCAGGACCGCATCCTGGAGATGAAGGTGATGGAGTTCTTCATG
AAGGTGTACGGCTACAGGGGAAAGCACCTGGGCGGAAGCAGAAAGCCTGACGGCGCCATCTATACAGTGGGC
AGCCCCATCGATTACGGCGTGATCGTGGACACAAAGGCCTACAGCGGCGGCTACAATCTGCCTATCGGCCAG
GCCGACGAGATGGAGAGATACGTGGAGGAGAACCAGACCCGGGATAAGCACCTCAACCCCAACGAGTGGTGG
AAGGTGTACCCTAGCAGCGTGACCGAGTTCAAGTTCCTGTTCGTGAGCGGCCACTTCAAGGGCAACTACAAG
GCCCAGCTGACCAGGCTGAACCACATCACCAACTGCAATGGCGCCGTGCTGAGCGTGGAGGAGCTGCTGATC
GGCGGCGAGATGATCAAAGCCGGCACCCTGACACTGGAGGAGGTGCGGCGCAAGTTCAACAACGGCGAGATC
AACTTCAGATCTTGATAA

MDYKDHDGDYKDHDIDYKDDDDKMAPKKKRKVGIHGVPAAMAERPFQCRICMRNFSEKRGLLNHIRTHTGE
KPFACDICGRKFASGAGLWQHTKIHTGSQKPFQCRICMRNFSRKAHLVDHIRTHTGEKPFACDICGRKFAA
RAGLWQHTKIHTGSQKPFQCRICMRNFSRKANLYNHIRTHTGEKPFACDICGRKFASQSGLCHHTKIHLRG
SQLVKSELEEKKSELRHKLKYVPHEYIELIEIARNSTQDRILEMKVMEFFMKVYGYRGKHLGGSRKPDGAI
YTVGSPIDYGVIVDTKAYSGGYNLPIGQADEMERYVEENQTRDKHLNPNEWWKVYPSSVTEFKFLFVSGHF
KGNYKAQLTRLNHITNCNGAVLSVEELLIGGEMIKAGTLTLEEVRRKFNNGEINFRS

>46702-CKKR
ATGGACTACAAAGACCATGACGGTGATTATAAAGATCATGACATCGATTACAAGGATGACGATGACAAGATG
GCCCCCAAGAAGAAGAGGAAGGTCGGCATTCATGGGGTACCCGCCGCTATGGCTGAGAGACCCTTTCAGTGT
CGCATCTGTATGCGGAACTTTTCCGCCCAGAGCAACCTGCTGAGACATATCCGCACACACACTGGAGAAAAG
CCTTTCGCATGTGACATTTGCGGACGCAAGTTTGCTAGAAAGCCCGACCTGGTGAGACACACTAAAATCCAT
ACAGGGTCCCAGAAACCATTCCAATGTAGGATTTGCATGCGCAATTTCTCTAGAAAGGACAACCTGAGAGAC
CACATTAGGACTCATACAGGGGAGAAACCATTCGCTTGCGATATCTGTGGGAGGAAATTCGCACAGAAGATC
AACCTGAACCAGCATACAAAGATTCACCTGAGAGGATCCCAGCTGGTGAAGAGCGAGCTGGAGGAGAAGAAG
TCCGAGCTGCGGCACAAGCTGAAGTACGTGCCCCACGAGTACATCGAGCTGATCGAGATCGCCAGGAACAGC
ACCCAGGACCGCATCCTGGAGATGAAGGTGATGGAGTTCTTCATGAAGGTGTACGGCTACAGGGGAAAGCAC
CTGGGCGGAAGCAGAAAGCCTGACGGCGCCATCTATACAGTGGGCAGCCCCATCGATTACGGCGTGATCGTG
GACACAAAGGCCTACAGCGGCGGCTACAATCTGCCTATCGGCCAGGCCGACGAGATGCAGAGATACGTGAAG
GAGAACCAGACCCGGAATAAGCACATCAACCCCAACGAGTGGTGGAAGGTGTACCCTAGCAGCGTGACCGAG
TTCAAGTTCCTGTTCGTGAGCGGCCACTTCAAGGGCAACTACAAGGCCCAGCTGACCAGGCTGAACCGCAAA
ACCAACTGCAATGGCGCCGTGCTGAGCGTGGAGGAGCTGCTGATCGGCGGCGAGATGATCAAAGCCGGCACC
CTGACACTGGAGGAGGTGCGGCGCAAGTTCAACAACGGCGAGATCAACTTCTGATAA

MDYKDHDGDYKDHDIDYKDDDDKMAPKKKRKVGIHGVPAAMAERPFQCRICMRNFSAQSNLLRHIRTHTGE
KPFACDICGRKFARKPDLVRHTKIHTGSQKPFQCRICMRNFSRKDNLRDHIRTHTGEKPFACDICGRKFAQ
KINLNQHTKIHLRGSQLVKSELEEKKSELRHKLKYVPHEYIELIEIARNSTQDRILEMKVMEFFMKVYGYR
GKHLGGSRKPDGAIYTVGSPIDYGVIVDTKAYSGGYNLPIGQADEMQRYVKENQTRNKHINPNEWWKVYPS
SVTEFKFLFVSGHFKGNYKAQLTRLNRKTNCNGAVLSVEELLIGGEMIKAGTLTLEEVRRKFNNGEINF

>46703-CKKR
ATGGACTACAAAGACCATGACGGTGATTATAAAGATCATGACATCGATTACAAGGATGACGATGACAAGATG
GCCCCCAAGAAGAAGAGGAAGGTCGGCATTCATGGGGTACCCGCCGCTATGGCTGAGAGACCCTTTCAGTGT
CGCATCTGTATGCGGAACTTTTCCCAGCTGTGCAACCTGATCAGACATATCCGCACACACACTGGAGAAAAG
CCTTTCGCATGTGACATTTGCGGACGCAAGTTTGCTCTGAAGAGCACCCTGGTGAACCACACTAAAATCCAT
ACAGGGTCCCAGAAACCATTCCAATGTAGGATTTGCATGCGCAATTTCTCTAGAAAGGACAACCTGAAGAGC
CACATTAGGACTCATACAGGGGAGAAACCATTCGCTTGCGATATCTGTGGGAGGAAATTCGCACAGAAGATC
AACCTGGTGAACCATACAAAGATTCACCTGAGAGGATCCCAGCTGGTGAAGAGCGAGCTGGAGGAGAAGAAG
TCCGAGCTGCGGCACAAGCTGAAGTACGTGCCCCACGAGTACATCGAGCTGATCGAGATCGCCAGGAACAGC

ACCCAGGACCGCATCCTGGAGATGAAGGTGATGGAGTTCTTCATGAAGGTGTACGGCTACAGGGGAAAGCAC
CTGGGCGGAAGCAGAAAGCCTGACGGCGCCATCTATACAGTGGGCAGCCCCATCGATTACGGCGTGATCGTG
GACACAAAGGCCTACAGCGGCGGCTACAATCTGCCTATCGGCCAGGCCGACGAGATGCAGAGATACGTGAAG
GAGAACCAGACCCGGAATAAGCACATCAACCCCAACGAGTGGTGGAAGGTGTACCCTAGCAGCGTGACCGAG
TTCAAGTTCCTGTTCGTGAGCGGCCACTTCAAGGGCAACTACAAGGCCCAGCTGACCAGGCTGAACCGCAAA
ACCAACTGCAATGGCGCCGTGCTGAGCGTGGAGGAGCTGCTGATCGGCGGCGAGATGATCAAAGCCGGCACC
CTGACACTGGAGGAGGTGCGGCGCAAGTTCAACAACGGCGAGATCAACTTCTGATAA

MDYKDHDGDYKDHDIDYKDDDDKMAPKKKRKVGIHGVPAAMAERPFQCRICMRNFSQLCNLIRHIRTHTGE
KPFACDICGRKFALKSTLVNHTKIHTGSQKPFQCRICMRNFSRKDNLKSHIRTHTGEKPFACDICGRKFAQ
KINLVNHTKIHLRGSQLVKSELEEKKSELRHKLKYVPHEYIELIEIARNSTQDRILEMKVMEFFMKVYGYR
GKHLGGSRKPDGAIYTVGSPIDYGVIVDTKAYSGGYNLPIGQADEMQRYVKENQTRNKHINPNEWWKVYPS
SVTEFKFLFVSGHFKGNYKAQLTRLNRKTNCNGAVLSVEELLIGGEMIKAGTLTLEEVRRKFNNGEINF

>46704-CKKR
ATGGACTACAAAGACCATGACGGTGATTATAAAGATCATGACATCGATTACAAGGATGACGATGACAAGATG
GCCCCCAAGAAGAAGAGGAAGGTCGGCATTCATGGGGTACCCGCCGCTATGGCTGAGAGACCCTTCCAGTGC
AGAATCTGCATGAGAAACTTCAGCACCAAGCAGAACCTGCAGACCCACATCAGAACCCACACCGGCGAGAAG
CCCTTTGCCTGCGACATCTGCGGCAGAAAGTTCGCCACCCTGTTCAACCTGACCAGACACACCAAGATCCAC
ACCGGCAGCCAGAAGCCCTTTCAGTGTCGCATCTGTATGCGGAACTTTTCCGCCCAGAGCAACCTGCTGAGA
CATATCCGCACACACACTGGAGAAAAGCCTTTCGCATGTGACATTTGCGGACGCAAGTTTGCTAGAAAGCCC
GACCTGGTGAGACACACTAAAATCCATACAGGGTCCCAGAAACCATTCCAATGTAGGATTTGCATGCGCAAT
TTCTCTAGAAAGGACAACCTGAGAGACCACATTAGGACTCATACAGGGGAGAAACCATTCGCTTGCGATATC
TGTGGGAGGAAATTCGCACAGAAGATCAACCTGAACCAGCATACAAAGATTCACCTGAGAGGATCCCAGCTG
GTGAAGAGCGAGCTGGAGGAGAAGAAGTCCGAGCTGCGGCACAAGCTGAAGTACGTGCCCCACGAGTACATC
GAGCTGATCGAGATCGCCAGGAACAGCACCCAGGACCGCATCCTGGAGATGAAGGTGATGGAGTTCTTCATG
AAGGTGTACGGCTACAGGGGAAAGCACCTGGGCGGAAGCAGAAAGCCTGACGGCGCCATCTATACAGTGGGC
AGCCCCATCGATTACGGCGTGATCGTGGACACAAAGGCCTACAGCGGCGGCTACAATCTGCCTATCGGCCAG
GCCGACGAGATGCAGAGATACGTGAAGGAGAACCAGACCCGGAATAAGCACATCAACCCCAACGAGTGGTGG
AAGGTGTACCCTAGCAGCGTGACCGAGTTCAAGTTCCTGTTCGTGAGCGGCCACTTCAAGGGCAACTACAAG
GCCCAGCTGACCAGGCTGAACCGCAAAACCAACTGCAATGGCGCCGTGCTGAGCGTGGAGGAGCTGCTGATC
GGCGGCGAGATGATCAAAGCCGGCACCCTGACACTGGAGGAGGTGCGGCGCAAGTTCAACAACGGCGAGATC
AACTTCTGATAA

MDYKDHDGDYKDHDIDYKDDDDKMAPKKKRKVGIHGVPAAMAERPFQCRICMRNFSTKQNLQTHIRTHTGE
KPFACDICGRKFATLFNLTRHTKIHTGSQKPFQCRICMRNFSAQSNLLRHIRTHTGEKPFACDICGRKFAR
KPDLVRHTKIHTGSQKPFQCRICMRNFSRKDNLRDHIRTHTGEKPFACDICGRKFAQKINLNQHTKIHLRG
SQLVKSELEEKKSELRHKLKYVPHEYIELIEIARNSTQDRILEMKVMEFFMKVYGYRGKHLGGSRKPDGAI
YTVGSPIDYGVIVDTKAYSGGYNLPIGQADEMQRYVKENQTRNKHINPNEWWKVYPSSVTEFKFLFVSGHF
KGNYKAQLTRLNRKTNCNGAVLSVEELLIGGEMIKAGTLTLEEVRRKFNNGEINF

>46705-CKKR
ATGGACTACAAAGACCATGACGGTGATTATAAAGATCATGACATCGATTACAAGGATGACGATGACAAGATG
GCCCCCAAGAAGAAGAGGAAGGTCGGCATTCATGGGGTACCCGCCGCTATGGCTGAGAGACCCTTCCAGTGC
AGAATCTGCATGAGAAACTTCAGCACCAAGCAGAACCTGACCCACCACATCAGAACCCACACCGGCGAGAAG
CCCTTTGCCTGCGACATCTGCGGCAGAAAGTTCGCCAGCCTGTTCAACCTGAAGAGACACACCAAGATCCAC
ACCGGCAGCCAGAAGCCCTTTCAGTGTCGCATCTGTATGCGGAACTTTTCCCAGCTGTGCAACCTGATCAGA
CATATCCGCACACACACTGGAGAAAAGCCTTTCGCATGTGACATTTGCGGACGCAAGTTTGCTCTGAAGAGC
ACCCTGGTGAACCACACTAAAATCCATACAGGGTCCCAGAAACCATTCCAATGTAGGATTTGCATGCGCAAT
TTCTCTAGAAAGGACAACCTGAAGAGCCACATTAGGACTCATACAGGGGAGAAACCATTCGCTTGCGATATC
TGTGGGAGGAAATTCGCACAGAAGATCAACCTGGTGAACCATACAAAGATTCACCTGAGAGGATCCCAGCTG
GTGAAGAGCGAGCTGGAGGAGAAGAAGTCCGAGCTGCGGCACAAGCTGAAGTACGTGCCCCACGAGTACATC
GAGCTGATCGAGATCGCCAGGAACAGCACCCAGGACCGCATCCTGGAGATGAAGGTGATGGAGTTCTTCATG
AAGGTGTACGGCTACAGGGGAAAGCACCTGGGCGGAAGCAGAAAGCCTGACGGCGCCATCTATACAGTGGGC
AGCCCCATCGATTACGGCGTGATCGTGGACACAAAGGCCTACAGCGGCGGCTACAATCTGCCTATCGGCCAG

GCCGACGAGATGCAGAGATACGTGAAGGAGAACCAGACCCGGAATAAGCACATCAACCCCAACGAGTGGTGG
AAGGTGTACCCTAGCAGCGTGACCGAGTTCAAGTTCCTGTTCGTGAGCGGCCACTTCAAGGGCAACTACAAG
GCCCAGCTGACCAGGCTGAACCGCAAAACCAACTGCAATGGCGCCGTGCTGAGCGTGGAGGAGCTGCTGATC
GGCGGCGAGATGATCAAAGCCGGCACCCTGACACTGGAGGAGGTGCGGCGCAAGTTCAACAACGGCGAGATC
AACTTCTGATAA

MDYKDHDGDYKDHDIDYKDDDDKMAPKKKRKVGIHGVPAAMAERPFQCRICMRNFSTKQNLTHHIRTHTGE
KPFACDICGRKFASLFNLKRHTKIHTGSQKPFQCRICMRNFSQLCNLIRHIRTHTGEKPFACDICGRKFAL
KSTLVNHTKIHTGSQKPFQCRICMRNFSRKDNLKSHIRTHTGEKPFACDICGRKFAQKINLVNHTKIHLRG
SQLVKSELEEKKSELRHKLKYVPHEYIELIEIARNSTQDRILEMKVMEFFMKVYGYRGKHLGGSRKPDGAI
YTVGSPIDYGVIVDTKAYSGGYNLPIGQADEMQRYVKENQTRNKHINPNEWWKVYPSSVTEFKFLFVSGHF
KGNYKAQLTRLNRKTNCNGAVLSVEELLIGGEMIKAGTLTLEEVRRKFNNGEINF

>49347-CKKR
ATGGACTACAAAGACCATGACGGTGATTATAAAGATCATGACATCGATTACAAGGATGACGATGACAAGATG
GCCCCCAAGAAGAAGAGGAAGGTCGGCATTCATGGGGTACCCGCCGCTATGGCTGAGCGCCCATTCCAGTGT
CGAATCTGCATGCGTAACTTCAGTCGGAAGCAGTGCCTTCAGCGGCACATCCGCACCCACACCGGCGAGAAG
CCTTTTGCCTGTGACATTTGTGGGAGGAAGTTTGCCTGGCCGAACAGCCTGAAAGCGCATACCAAAATCCAT
ACAGGTTCCCAGAAACCGTTTCAATGCAGGATATGCATGCGTAACTTCAGTGACAGGAGCAACCTTACGAAG
CACATCCGCACCCACACCGGTGAAAAACCATTCGCATGCGATATCTGTGGGAGGAAGTTTGCCCATAAACAT
CATCTGACCGAGCATACCAAAATCCATACCGGCAGCCAGAAGCCATTTCAGTGCCGCATTTGCATGCGTAAC
TTCAGTTGGCCCAACAGCCTGAAGTACCACATCCGCACCCACACCGGCGAGAAGCCTTTTGCCTGTGACATT
TGTGGGAGGAAGTTTGCCGACAGAAGCGCCCTGATCAGACATACCAAAATCCATTTACGTGGATCCCAGCTG
GTGAAGAGCGAGCTGGAGGAGAAGAAGTCCGAGCTGCGGCACAAGCTGAAGTACGTGCCCCACGAGTACATC
GAGCTGATCGAGATCGCCAGGAACAGCACCCAGGACCGCATCCTGGAGATGAAGGTGATGGAGTTCTTCATG
AAGGTGTACGGCTACAGGGGAAAGCACCTGGGCGGAAGCAGAAAGCCTGACGGCGCCATCTATACAGTGGGC
AGCCCCATCGATTACGGCGTGATCGTGGACACAAAGGCCTACAGCGGCGGCTACAATCTGCCTATCGGCCAG
GCCGACGAGATGCAGAGATACGTGAAGGAGAACCAGACCCGGAATAAGCACATCAACCCCAACGAGTGGTGG
AAGGTGTACCCTAGCAGCGTGACCGAGTTCAAGTTCCTGTTCGTGAGCGGCCACTTCAAGGGCAACTACAAG
GCCCAGCTGACCAGGCTGAACCGCAAAACCAACTGCAATGGCGCCGTGCTGAGCGTGGAGGAGCTGCTGATC
GGCGGCGAGATGATCAAAGCCGGCACCCTGACACTGGAGGAGGTGCGGCGCAAGTTCAACAACGGCGAGATC
AACTTCTGATAA

MDYKDHDGDYKDHDIDYKDDDDKMAPKKKRKVGIHGVPAAMAERPFQCRICMRNFSRKQCLQRHIRTHTGE
KPFACDICGRKFAWPNSLKAHTKIHTGSQKPFQCRICMRNFSDRSNLTKHIRTHTGEKPFACDICGRKFAH
KHHLTEHTKIHTGSQKPFQCRICMRNFSWPNSLKYHIRTHTGEKPFACDICGRKFADRSALIRHTKIHLRG
SQLVKSELEEKKSELRHKLKYVPHEYIELIEIARNSTQDRILEMKVMEFFMKVYGYRGKHLGGSRKPDGAI
YTVGSPIDYGVIVDTKAYSGGYNLPIGQADEMQRYVKENQTRNKHINPNEWWKVYPSSVTEFKFLFVSGHF
KGNYKAQLTRLNRKTNCNGAVLSVEELLIGGEMIKAGTLTLEEVRRKFNNGEINF

>46710-CKKR
ATGGACTACAAAGACCATGACGGTGATTATAAAGATCATGACATCGATTACAAGGATGACGATGACAAGATG
GCCCCCAAGAAGAAGAGGAAGGTCGGCATTCATGGGGTACCCGCCGCTATGGCTGAGCGCCCATTCCAGTGT
CGAATCTGCATGCGTAACTTCAGTTTGAAGGGGAACCTTTTGAAGCACATCCGCACCCACACCGGCGAGAAG
CCTTTTGCCTGTGACATTTGTGGGAGGAAGTTTGCCATGAAACATCATCTGACCGAACATACCAAAATCCAT
ACAGGTTCCCAGAAACCGTTTCAATGCAGGATATGTATGCGTAACTTCAGTTGGCCGAACAGCCTGAAATAT
CACATCCGCACCCACACCGGCGAGAAGCCTTTTGCCTGTGACATTTGTGGGAGGAAGTTTGCCGACCGCAGC
GCGCTCATCCGGCATACCAAAATCCATCTGCGCGGATCCCAGCTGGTGAAGAGCGAGCTGGAGGAGAAGAAG
TCCGAGCTGCGGCACAAGCTGAAGTACGTGCCCCACGAGTACATCGAGCTGATCGAGATCGCCAGGAACAGC
ACCCAGGACCGCATCCTGGAGATGAAGGTGATGGAGTTCTTCATGAAGGTGTACGGCTACAGGGGAAAGCAC
CTGGGCGGAAGCAGAAAGCCTGACGGCGCCATCTATACAGTGGGCAGCCCCATCGATTACGGCGTGATCGTG
GACACAAAGGCCTACAGCGGCGGCTACAATCTGCCTATCGGCCAGGCCGACGAGATGCAGAGATACGTGAAG
GAGAACCAGACCCGGAATAAGCACATCAACCCCAACGAGTGGTGGAAGGTGTACCCTAGCAGCGTGACCGAG
TTCAAGTTCCTGTTCGTGAGCGGCCACTTCAAGGGCAACTACAAGGCCCAGCTGACCAGGCTGAACCGCAAA

ACCAACTGCAATGGCGCCGTGCTGAGCGTGGAGGAGCTGCTGATCGGCGGCGAGATGATCAAAGCCGGCACC
CTGACACTGGAGGAGGTGCGGCGCAAGTTCAACAACGGCGAGATCAACTTCTGATAA

MDYKDHDGDYKDHDIDYKDDDDKMAPKKKRKVGIHGVPAAMAERPFQCRICMRNFSLKGNLLKHIRTHTGE
KPFACDICGRKFAMKHHLTEHTKIHTGSQKPFQCRICMRNFSWPNSLKYHIRTHTGEKPFACDICGRKFAD
RSALIRHTKIHLRGSQLVKSELEEKKSELRHKLKYVPHEYIELIEIARNSTQDRILEMKVMEFFMKVYGYR
GKHLGGSRKPDGAIYTVGSPIDYGVIVDTKAYSGGYNLPIGQADEMQRYVKENQTRNKHINPNEWWKVYPS
SVTEFKFLFVSGHFKGNYKAQLTRLNRKTNCNGAVLSVEELLIGGEMIKAGTLTLEEVRRKFNNGEINF

>46711-NELD
ATGGACTACAAAGACCATGACGGTGATTATAAAGATCATGACATCGATTACAAGGATGACGATGACAAGATG
GCCCCCAAGAAGAAGAGGAAGGTCGGCATCCACGGGGTACCCGCCGCTATGGCTGAGAGACCCTTTCAGTGT
CGCATCTGTATGCGGAACTTTTCCACCAAGTGGAACCTGACCCAGCATATCCGCACACACACTGGAGAAAAG
CCTTTCGCATGTGACATTTGCGGACGCAAGTTTGCTTTCAAGAGCAACCTGACCAACCACACTAAAATCCAT
ACAGGGTCCCAGAAACCATTCCAATGTAGGATTTGCATGCGCAATTTCTCTAGAAAGGCCCACCTGGTGAAC
CACATTAGGACTCATACAGGGGAGAAACCATTCGCTTGCGATATCTGTGGGAGGAAATTCGCAGACAGAGCC
AACCTGATCCACCATACAAAGATTCACCTGAGAGGATCCCAGCTGGTGAAGAGCGAGCTGGAGGAGAAGAAG
TCCGAGCTGCGGCACAAGCTGAAGTACGTGCCCCACGAGTACATCGAGCTGATCGAGATCGCCAGGAACAGC
ACCCAGGACCGCATCCTGGAGATGAAGGTGATGGAGTTCTTCATGAAGGTGTACGGCTACAGGGGAAAGCAC
CTGGGCGGAAGCAGAAAGCCTGACGGCGCCATCTATACAGTGGGCAGCCCCATCGATTACGGCGTGATCGTG
GACACAAAGGCCTACAGCGGCGGCTACAATCTGCCTATCGGCCAGGCCGACGAGATGGAGAGATACGTGGAG
GAGAACCAGACCCGGGATAAGCACCTCAACCCCAACGAGTGGTGGAAGGTGTACCCTAGCAGCGTGACCGAG
TTCAAGTTCCTGTTCGTGAGCGGCCACTTCAAGGGCAACTACAAGGCCCAGCTGACCAGGCTGAACCACATC
ACCAACTGCAATGGCGCCGTGCTGAGCGTGGAGGAGCTGCTGATCGGCGGCGAGATGATCAAAGCCGGCACC
CTGACACTGGAGGAGGTGCGGCGCAAGTTCAACAACGGCGAGATCAACTTCAGATCTTGATAA

MDYKDHDGDYKDHDIDYKDDDDKMAPKKKRKVGIHGVPAAMAERPFQCRICMRNFSTKWNLTQHIRTHTGE
KPFACDICGRKFAFKSNLTNHTKIHTGSQKPFQCRICMRNFSRKAHLVNHIRTHTGEKPFACDICGRKFAD
RANLIHHTKIHLRGSQLVKSELEEKKSELRHKLKYVPHEYIELIEIARNSTQDRILEMKVMEFFMKVYGYR
GKHLGGSRKPDGAIYTVGSPIDYGVIVDTKAYSGGYNLPIGQADEMERYVEENQTRDKHLNPNEWWKVYPS
SVTEFKFLFVSGHFKGNYKAQLTRLNHITNCNGAVLSVEELLIGGEMIKAGTLTLEEVRRKFNNGEINFRS

>46713-NELD
ATGGACTACAAAGACCATGACGGTGATTATAAAGATCATGACATCGATTACAAGGATGACGATGACAAGATG
GCCCCCAAGAAGAAGAGGAAGGTCGGCATCCACGGGGTACCCGCCGCTATGGCTGAGAGACCCTTCCAGTGC
AGAATCTGCATGAGAAACTTCAGCGACAGAAGCAACCTGAGAGCCCACATCAGAACCCACACCGGCGAGAAG
CCCTTTGCCTGCGACATCTGCGGCAGAAAGTTCGCCAGAAAGTTCACCCTGACCAACCACACCAAGATCCAC
ACCGGCAGCCAGAAGCCCTTTCAGTGTCGCATCTGTATGCGGAACTTTTCCACCAAGTGGAACCTGACCCAG
CATATCCGCACACACACTGGAGAAAAGCCTTTCGCATGTGACATTTGCGGACGCAAGTTTGCTTTCAAGAGC
AACCTGACCAACCACACTAAAATCCATACAGGGTCCCAGAAACCATTCCAATGTAGGATTTGCATGCGCAAT
TTCTCTAGAAAGGCCCACCTGGTGAACCACATTAGGACTCATACAGGGGAGAAACCATTCGCTTGCGATATC
TGTGGGAGGAAATTCGCAGACAGAGCCAACCTGATCCACCATACAAAGATTCACCTGAGAGGATCCCAGCTG
GTGAAGAGCGAGCTGGAGGAGAAGAAGTCCGAGCTGCGGCACAAGCTGAAGTACGTGCCCCACGAGTACATC
GAGCTGATCGAGATCGCCAGGAACAGCACCCAGGACCGCATCCTGGAGATGAAGGTGATGGAGTTCTTCATG
AAGGTGTACGGCTACAGGGGAAAGCACCTGGGCGGAAGCAGAAAGCCTGACGGCGCCATCTATACAGTGGGC
AGCCCCATCGATTACGGCGTGATCGTGGACACAAAGGCCTACAGCGGCGGCTACAATCTGCCTATCGGCCAG
GCCGACGAGATGGAGAGATACGTGGAGGAGAACCAGACCCGGGATAAGCACCTCAACCCCAACGAGTGGTGG
AAGGTGTACCCTAGCAGCGTGACCGAGTTCAAGTTCCTGTTCGTGAGCGGCCACTTCAAGGGCAACTACAAG
GCCCAGCTGACCAGGCTGAACCACATCACCAACTGCAATGGCGCCGTGCTGAGCGTGGAGGAGCTGCTGATC
GGCGGCGAGATGATCAAAGCCGGCACCCTGACACTGGAGGAGGTGCGGCGCAAGTTCAACAACGGCGAGATC
AACTTCAGATCTTGATAA

MDYKDHDGDYKDHDIDYKDDDDKMAPKKKRKVGIHGVPAAMAERPFQCRICMRNFSDRSNLRAHIRTHTGE
KPFACDICGRKFARKFTLTNHTKIHTGSQKPFQCRICMRNFSTKWNLTQHIRTHTGEKPFACDICGRKFAF

<u>KSNLTN</u>HTKIHTGSQKPFQCRICMRNFS<u>RKAHLVN</u>HIRTHTGEKPFACDICGRKFA<u>DRANLIH</u>HTKIHLRG
SQLVKSELEEKKSELRHKLKYVPHEYIELIEIARNSTQDRILEMKVMEFFMKVYGYRGKHLGGSRKPDGAI
YTVGSPIDYGVIVDTKAYSGGYNLPIGQADEMERYVEENQTRDKHLNPNEWWKVYPSSVTEFKFLFVSGHF
KGNYKAQLTRLNHITNCNGAVLSVEELLIGGEMIKAGTLTLEEVRRKFNNGEINFRS
